# Development and validation of a new high-throughput method to investigate the clonality of HTLV-1-infected cells based on provirus integration sites

**DOI:** 10.1186/gm568

**Published:** 2014-06-27

**Authors:** Sanaz Firouzi, Yosvany López, Yutaka Suzuki, Kenta Nakai, Sumio Sugano, Tadanori Yamochi, Toshiki Watanabe

**Affiliations:** 1 Department of Medical Genome Science, Graduate School of Frontier Sciences, The University of Tokyo, 4-6-1 Shirokanedai, Minato-ku, Tokyo 108-8639, Japan; 2 Department of Computational Biology, Graduate School of Frontier Sciences, The University of Tokyo, 5-1-5 Kashiwanoha, Kashiwa-shi, Chiba-ken 277-8561, Japan; 3 Human Genome Center, The Institute of Medical Science, The University of Tokyo, 4-6-1 Shirokanedai, Minato-ku, Tokyo 108-8639, Japan

## Abstract

Transformation and clonal proliferation of T-cells infected with human T-cell leukemia virus type-I (HTLV-1) cause adult T-cell leukemia. We took advantage of next-generation sequencing technology to develop and internally validate a new methodology for isolating integration sites and estimating the number of cells in each HTLV-1-infected clone (clone size). Initial analysis was performed with DNA samples from infected individuals. We then used appropriate controls with known integration sites and clonality status to confirm the accuracy of our system, which indeed had the least errors among the currently available techniques. Results suggest potential clinical and biological applications of the new method.

## Background

It has been more than 30 years since human T-cell leukemia virus type-I (HTLV-1) was shown to be the causative agent of adult T-cell leukemia (ATL) [1,2]. However, understanding the true nature of the multiple leukemogenic events [3] that are essential for this aggressive transformation remains elusive [4-9]. Although approximately 5% of HTLV-1-infected individuals develop ATL after a long latency period, the majority remain asymptomatic carriers (ACs) throughout their lifetimes. However, there are not enough clear determinants to distinguish between individuals who eventually develop ATL and those who remain as ACs [10,11]. To discover the factors associated with disease development, long-term prospective studies have assessed the correlation between disease outcome and proviral load (PVL), that is, the percentage of infected cells among the total peripheral blood mononuclear cells (PBMCs) [10-12]. The ‘Joint Study on Predisposing Factors of ATL Development’ (JSPFAD) [13] showed that a PVL higher than 4% is one of the indications of risk for progression to ATL [10]. Although an elevated PVL is currently the best characterized factor associated with a high risk of ATL development, a high PVL alone is not sufficient for disease prediction, suggesting the need to discover additional predictive factors [10,11].

Because ATL is a malignancy caused by HTLV-1 infection, both the integration of provirus into the host genome and the clonal expansion of infected cells are highly critical leukemogenic events [6,7,14,15]. Although many studies have addressed these aspects, the mechanism of HTLV-1 clonal expansion has not been elucidated [15-35]. Accurate monitoring for changes in clonality occurring before, during, and after ATL development is of great interest and of major clinical significance not only to clarify the underlying mechanisms but also to discover reliable predictive biomarkers for disease progression.

A broad range of evidence strongly supports that most neoplasms are composed of clonally expanded cell populations [36-38]. Owing to its biological significance, the concept of clonal expansion in cancer biology has been investigated using a variety of approaches in many tumor types [36-39], including ATL [6,15,16,18-20,22,24,29-32]. Clonal proliferation of HTLV-1-infected cells was first detected as monoclonal-derived bands by southern blotting [33]. Early studies found that monoclonal integration of HTLV-1 is a hallmark of ATL cells [16]. Furthermore, it was suggested that detecting a monoclonal band is useful for diagnosis and is associated with a high risk of ATL development [29,30]. Subsequent PCR-based methods included inverse PCR, linker-mediated PCR, and inverse long PCR, which enabled analysis of samples with clonality below the detection threshold of southern blotting [17,25,31,34]. Based on the observed banding patterns, the clonality of the samples was described as having undergone monoclonal, oligoclonal, or polyclonal expansion. Such PCR-based analyses revealed that, in addition to a monoclonal proliferation of infected cells, a monoclonal or polyclonal proliferation occurs even in non-malignant HTLV-1 carriers [31,35]. Moreover, considering the stability of the HTLV-1 proviral sequence, it was hypothesized that maintaining a high PVL is achieved by persistent clonal proliferation of infected cells *in vivo*[25]. This hypothesis was further supported by the detection of a particular HTLV-1 clone in the same carrier over the course of several years [18]. Two Miyazaki cohort studies focused on the maintenance and establishment of clonal expansion: Okayama *et al.* analyzed the maintenance of a pre-leukemic clone in an AC state several years prior to ATL onset [19], and Tanaka *et al.* assessed the establishment of clonal expansion by comparing the clonality status of long-term carriers with that of seroconverters. They showed that some of the clones from long-term carriers were stable and large enough to be consistently detectable by inverse long PCR; however, those from seroconverters were unstable and rarely detectable over time [20].

Knowledge provided by conventional studies has shed light on the next challenges worthy of further investigation. Owing to technical hurdles, however, previous studies isolated small numbers of integration sites from highly abundant clones and detected low abundant clones in a non-reproducible manner [22,34]. Furthermore, conventional techniques could not provide adequate information regarding the number of infected cells in each clone (clone size) [22]. To effectively track and monitor HTLV-1 clonal composition and dynamics, we considered devising a new method that would not only enable the high-throughput isolation of integration sites but also provide an accurate measurement of clone size.

PCR is a necessary step for the integration site isolation and clonality analysis. However, bias in the amplification of DNA fragments (owing to issues such as extreme fragment length and high GC content) is intrinsic to any PCR-based method [40-45]. Different fragment amplification efficiencies make it difficult to calculate the amount of starting DNA (the original distribution of template DNA) from PCR products. Hence, estimating HTLV-1 clonal abundance, which requires calculating the number of starting DNA fragments, is only achievable by avoiding the PCR bias.

Recently, Bangham’s research group analyzed HTLV-1 clonality and integration site preference by a high-throughput method [22]. In the method developed by Gillet *et al.*, clone sizes were estimated using length of DNA fragments (shear sites generated by sonication) as a strategy for removing PCR bias [22]. Owing to the limited variation in DNA fragment size observed with shearing, the probability of generating starting fragments of the same lengths is high, leading to a nonlinear relationship between fragment length and clone size [22,46]. Therefore, Gillet *et al.* used a calibration curve to statistically correct the shear site data [22]. Later, Berry *et al.* introduced a statistical approach, and further addressed the difficulties of estimating clone size from shear site data [46]. Their approach estimates the size of small clones with little error, but estimates for larger clones have greater error [46]. A parameter adopted from the Gini coefficient [47,48] and termed the oligoclonality index was used to describe the size and distribution of HTLV-1 clones [22]. It has been demonstrated that the oligoclonality index differs between malignant and non-malignant HTLV-1 infections, and also a high PVL of HTLV-1-associated myelopathy is due to cells harboring large numbers of unique integration sites [22]. Furthermore, genome-wide integration site profiling of clinical samples revealed that the abundance of a given clone *in vivo* correlates with the features of the flanking host genome [22,24]; although there was not a specific hotspot, HTLV-1 more frequently integrated in transcriptionally active regions of the host genome [22,24]. These findings further clarified the characteristics of HTLV-1 integration sites, and strongly suggested the importance of HTLV-1 clonal expansion *in vivo*.

Here we introduce a method that overcomes many of the limitations of currently available methods. Taking advantage of next-generation sequencing (NGS) technology, nested-splinkerette PCR, and a tag system, we designed a new high-throughput method that enables specific isolation of HTLV-1 integration sites and, most importantly, allows for the quantification of clonality not only from the major clones and high-PVL samples but also from low-abundance clones (minor clones) and samples with low PVLs. Moreover, we conducted comprehensive internal validation experiments to assess the effectiveness and accuracy of our new methodology. A preliminary validation was conducted by analyzing DNA samples from HTLV-1-infected individuals with different PVLs and disease status. Subsequently, an internal validation was performed that included an appropriate control with known integration sites and clonality patterns. We present our methodology, which illustrates that employing the tag system is effective for improving quantification of clonal abundance.

## Methods

Our clonality analysis method included two main aspects: (1) wet experiments, and (2) *in silico* analysis (Additional file 1: Figure S1). A general explanation of materials and methods is provided here, and detailed protocols of the wet experiments are included in Additional file 1: Notes. The *in silico* analysis is further described in Results and discussion.

NGS data have been deposited in the Sequence Read Archive of NCBI with access number of (SRP038906).

### Wet experiments

#### ***Biological samples: specimens and cell lines***

Specimens: In total five clinical samples were provided by a biomaterial bank of HTLV-1 carriers, JSPFAD [13,49]. The clinical samples were a part of those collected with an informed consent as a collaborative project of JSPFAD. The project was approved by the Institute of Medical Sciences, the University of Tokyo (IMSUT) Human Genome Research Ethics Committee. Information about the disease status of samples was obtained from JSPFAD database in which HTLV-1-infected individuals were diagnosed based on the Shimoyama criteria [50]. In brief, genomic DNA from PBMCs was isolated using a QIAGEN Blood kit. PVLs were measured by real-time PCR using the ABI PRISM 7000 Sequence Detection System as described in [10].

Cell lines: An IL2-dependent TL-Om1 cell line [51] was maintained in RPMI 1640 medium supplemented with 10% heat-inactivated fetal calf serum (GIBCO), 1% penicillin-streptomycin (GIBCO), and 10 ng/mL IL2 (R&D systems). The same conditions as those of patient samples were used to extract DNA and measure PVL.

#### ***Illumina-specific library construction***

We employed a library preparation protocol specifically designed to isolate HTLV-1 integration sites. The final products in the library that we generated contained all the specific sequences necessary for the Illumina HiSeq 2000 platform (Additional file 1: Figure S2). These products included a 5′-flow cell binding sequence, a region compatible with read-1 sequencing primer, 5-bp random nucleotides, 5-bp known barcodes for multiplexing samples, HTLV-1 long terminal repeat (LTR), human or HTLV-1 genomic DNA, a region compatible with read-2 and read-3 sequencing primers, 8-bp random tags, and a 3′-flow cell binding sequence from 5′ to 3′, respectively (Additional file 1: Figure S2B).

Incorporating the 5-bp random nucleotides downstream of the region compatible with the read-1 sequencing primer was critical and resulted in high-quality sequence data. We used a library designed without the first 5-bp of random nucleotides as input for the HiSeq 2000 sequencer in our first samples (S-1, S-2, S-3, and S-4). Because all fragments began with the same LTR sequence, clusters generated in the flow cells could not be differentiated appropriately. These samples resulted in low-quality sequence data (see Additional file 1: Notes). Designing the first 5-bp randomly resulted in high-quality sequence data for the remaining samples because clusters were differentiated with no problem during the first five cycles of sequencing (data not shown).

Our library construction pipeline comprised the following four steps (Additional file 1: Figure S2) (Additional file 1: Notes):

**Figure 1:**
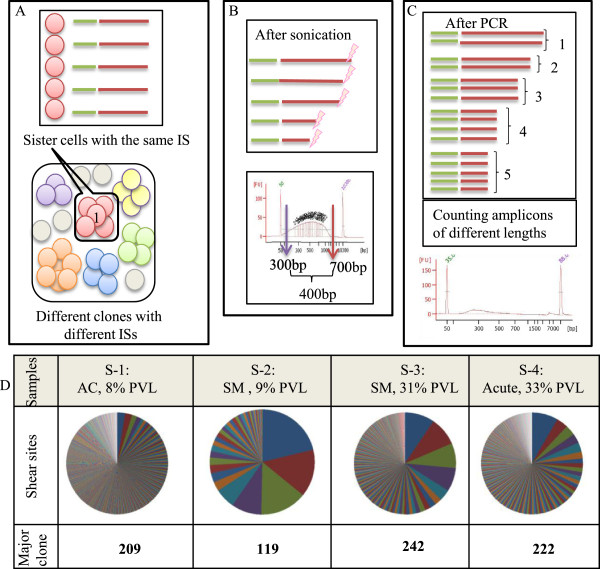
**Estimating clone size by ‘shear sites’.** Also see Additional file 2: Figure S2 for a simple image from an integration site and its shear sites. **(A)** Depicted is the complex population of uninfected cells (grey circles) together with infected clones (circles of different colors). A clone is shown as a group of sister cells (circles of the same color) having the same integration site (IS). Different clones are distinguishable based on differing integration sites, and thus the number of integration sites represents the number of infected clones. For example, the six different unique integration sites refer to six unique clones. **(B)** Genomic DNA fragmented by sonication generates random shear sites (fragments of different length). Fragment size, measured by an Agilent Bioanalyzer, ranged from 300 to 700 bp. This size range can theoretically provide approximately 400 variations. **(C)** The size distribution of fragments decreased following amplification by integration-site-specific PCR. From the deep sequencing data, the original number of starting fragments could be estimated by removing PCR duplicates and counting fragments with different lengths. For example, five different lengths of PCR amplicons represent five infected sister cells. **(D)** We analyzed four samples, including (S-1: asymptomatic carrier (AC), (8% PVL)), (S-2: smoldering (SM), (9% PVL)), (S-3: smoldering, (31% PVL)), and (S-4: acute, (33% PVL)). Using our method, the clone sizes were quantified by considering only shear sites. The first major clone (the largest clone) of each sample was mapped to (chr 11-41829319 (+)), (chr 15: 59364370 (+)), (chr 4-563543 (-)), and (chr X - 83705328 (-)), respectively. The shear site variations of each major clone were 209, 119, 242, and 222, respectively. Different colors on the pie graphs indicate different integration sites, and the size of each piece represents the clone size.

(1) DNA isolation: DNA was extracted as described above, and the concentration of extracted DNA was measured with a NanoDrop 2000 spectrophotometer (Thermo Scientific). We recommend using 10 μg of DNA as the starting material. However, in practice there are some rare clinical samples with limited DNA available. In order to be able to handle those samples, the method was also optimized for 5 μg and 2 μg of starting DNA.(2) Fragmentation: According to the protocol provided in Supplementary Notes, the starting template DNA was sheared by sonication. The resulting fragments represented a size range of 300 to 700 bp as checked by an Agilent 2100 Bioanalyzer and DNA 7500 kit (Figure 1B).

(3) Pre-PCR manipulations: Four steps of end repair, A-tailing, adaptor ligation, and size selection were performed as described in Additional file 1: Notes.

(4) PCR: To amplify the junction between the genome and the viral insert, we used nested-splinkerette PCR (a variant of ligation-mediated PCR [52,53]) (Additional file 1: Figure S2). We confirmed that the technique specifically amplifies HTLV-1 integration sites; since there was no non-specific amplification neither from human endogenous retroviruses nor from an exogenous retrovirus such as HIV (see Additional file 1: Table S1 and Additional file 2: Figure S1).

Information on oligonucleotides, including adaptors and primers, and the LTR and HTLV-1 reference sequences [54] are provided in Additional file 1: Table S1. The final PCR products were sequenced using the HiSeq 2000 platform.

### *In silico* analysis

Raw sequencing data were processed according to the workflow described in the Results and discussion section. The initial forward read (100-bp) was termed Read-1 and the reverse read (100-bp) was termed Read-3 and an index read (8-bp) was termed Read-2. In brief, analysis programs were written in Perl language and run on a supercomputer system provided by The University of Tokyo’s Human Genome Center at The Institute of Medical Science [55]. The sequencing output was check for quality using the FastQC tool [56]. The regions corresponding to the LTR and HTLV-1 genome were subjected to a blast search against the reference sequences described in Additional file 1: Table S1. Following isolation of the integration sites, the flanking human sequences were mapped to the human genome (hg19) (the UCSC genome browser [57]) by Bowtie 1.0.0 [58]. The final processed data included information about shear sites (R1R3), tags (R1R2), and a combination of tags and shear sites (R1R2R3). Fitting the data to the zero truncated Poisson distribution for retrieving correlation coefficients were done by the R-package ‘gamlss.tr’ [59]. The Gini coefficient was calculated by StatsDirect medical statistics software [60].

## Results and discussion

### General concepts

We originally designed our method to overcome the limitations of conventional techniques [31,34] and to make improvements in the only existing high-throughput method [22]. In general, our method includes two main sets of wet experiments and an *in-silico* analysis. We used genomic DNA as the starting material to prepare an appropriate library for Illumina sequencing. Subsequently, deep-sequencing data were analyzed by a supercomputer. The resulting information represents the clonality status of each sample (Additional file 1: Figure S1).

There are complex populations of infected clones and uninfected cells in a given HTLV-1 infected individual. High-throughput clonality analysis requires monitoring two main characteristics of clones: HTLV-1 integration sites and the number of infected cells in each clone (clone size). Each HTLV-1-infected cell naturally harbors only a single integration site [23]. Therefore, the number of detected unique integration sites corresponds to the number of infected clones. Based on our analysis, which is consistent with the data of Gillet *et al*. [22], employing high-sensitivity deep sequencing allowed for the isolation of a large number of unique integration sites (UISs), including samples with low PVLs (Figure 1). We analyzed four samples from HTLV-1-infected individuals with different PVLs, disease status, and expected clonality patterns. The samples include S-1: AC (8% PVL); S-2: smoldering ATL (SM) (9% PVL); S-3: SM (31% PVL); and S-4: acute ATL (33% PVL). Based on the final optimized conditions, 1030, 39, 265, and 384 UISs were isolated from each sample, respectively (Figure 1).

The most challenging aspect of our clonality analysis was estimating the number of infected cells in each clone. Although a necessary step in the analysis, PCR introduces a bias in the frequency of starting DNA material [40-45]. Because amplification causes significant changes in the initial frequency of starting materials, PCR products cannot be used directly to estimate the amount of the starting DNA material. To overcome this problem, we needed to manipulate DNA fragments to make them unique prior to PCR amplification. Thus, if each DNA fragment could be marked with a unique feature, it would then be possible to calculate its frequency based on the frequency of that unique feature. When a single unique stretch of DNA is amplified by PCR, the resulting product is a cluster of identical fragments termed PCR duplicates. Therefore, to estimate the frequency of starting DNA fragments, one should count the number of clusters with unique features. The remaining technical question then becomes how to mark the starting DNA prior to PCR amplification. In the following section, we compare and discuss two main strategies, namely (1) shear sites and (2) a tag system, which enable DNA fragments to be uniquely marked.

### Estimating the size of clones by shear sites

The first strategy, described by Gillet *et al.*, relies on shearing DNA by sonication, resulting in fragments of random length [22]. Sonication-derived shear sites were thus used as a distinguishing feature to make fragments unique prior to PCR. Clone sizes were then estimated by statistical approaches [22,46].

To directly assess the effectiveness of the shear site strategy, we analyzed the clonality of the aforementioned clinical samples (S-1, S-2, S-3, and S-4). Genomic DNA was cleaved by sonication with fragments in the 300- to 700-bp range, theoretically providing approximately 400 possible variations in fragment size (Figure 1A and B). Following library construction, however, the final product represented smaller size ranges, implying a relatively limited number of variations (Figure 1C). Finally, the number of PCR amplicons with unique shear sites was retrieved from deep-sequencing data. See Additional file 2: Figure S2 for a simple image from an integration site and its shear sites. The data obtained from the shear site experiments were not fitted to calibration curves or statistical treatments, which were used by Gillet *et al.* and Berry *et al.*, respectively (See Additional file 1: Notes) [22,46]. For clarity, only the information relating to the major clone of each sample is provided in Figure 1D. The shear-site variations of the major clone were 209, 119, 242, and 222 for samples S-1 through S-4, respectively. Even in the case of control samples with 100% PVLs, the shear sites did not provide more than 225 variations (see Validation of the methodology). However, it was expected that samples with differing PVLs and disease status would harbor varying numbers of sister cells, at least in their major clones. Similar variations of shear sites were observed in major clones of AC, SM, and acute samples. These data suggest that, because the number of sister cells in each clone exceeded the shear site variations, the size of the clones was underestimated (Figure 1). This is most problematic in the case of large clones and leads to an underestimation of the clone size.

### Measuring the size of clones by the tag system

**Figure 2:**
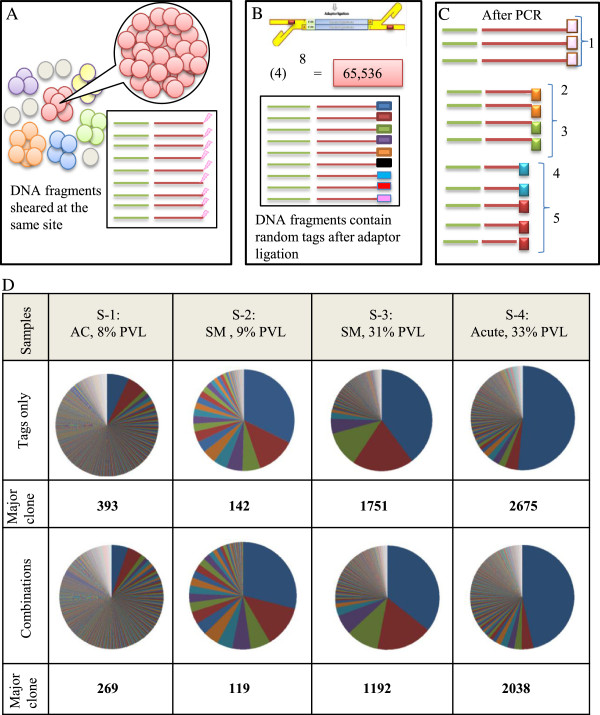
**Measuring clone size using the tag system. (A)** The depiction above shows that shear site variations are not able to cover all sister cells in large clones. As the number of the sister cells in a given clone increases, the probability of DNA shearing at the same site increases. **(B)** Prior to PCR, we incorporated 8-bp random tags into each DNA fragment to uniquely mark them. Random tags could theoretically provide approximately 65,536 variations. The number of potential variations is expected to amply cover large numbers of the sister cells. **(C)** The tag information was used to remove PCR duplicates and to estimate the original number of starting fragments. If the fragments had the same shear sites but different tags, they were counted separately. For example, here five different combinations of tags and shear sites represent five infected cells. **(D)** Samples: S-1, S-2, S-3, and S-4 were analyzed by the final optimal condition (Bowtie parameters: -v 3 - - best, and filtering condition: (merging approach) JT-10). Clone size was measured by tags only or by the combination of shear sites and tags. The covered variations were (393,142, 1751, and 2675) and (269, 119, 1192, and 2038), respectively.

We developed an alternate strategy to remove PCR bias and to estimate starting DNA. We designed a tag system in which 8-bp random nucleotides are incorporated at the end of DNA fragments during adaptor ligation step. Each tag acts as a molecular barcode, which gives each DNA fragment a unique signature prior to PCR. Information on the frequency of observed tags from the deep-sequencing data can be used to remove the PCR duplicates and thereby estimate the original clonal abundance in the starting sample. Owing to their random design, the tags could theoretically provide approximately 65,536 variations. This degree of potential variation is expected to provide a unique tag for a large number of sister cells in each clone (Figure 2).

We analyzed samples S-1, S-2, S-3, and S-4 to assess the effectiveness of our tag system for estimating clone size. The major clone of each sample showed tag variations of 393, 142, 1751, and 2675, respectively (Figure 2D). Similar variations of tags and shear sites were observed in the largest clones of S-1 and S-2 ((shear sites *vs.* tags): (209 *vs.* 393) and (119 *vs.* 142)) (Figure 1D and Figure 2D). In all four samples, those variations were also similar in the minor clones of which the clone sizes did not exceed shear sites variations (approximately <200 variations) (See Additional file 1: Table S3 and Additional file 2: Table S1 for information on the ten largest clones). However, the variations covered by tags were significantly greater than those of shear sites, especially for large clones like those observed in the major clones of S-3 and S-4 ((shear sites *vs.* tags): (242 *vs.* 1751) and (222 *vs.* 2675)). The variations covered by tags and combinations were almost the same for all four samples ((tags *vs.* combinations): (393 *vs.* 296), (142 *vs.* 119), (1751 *vs.* 1192), and (2675 *vs.* 2038)).

Upon comparison of the tag system data with the shear site data, it was clear that both strategies yield essentially the same results when the size of clones is small enough to be covered by the number of shear site variations generated. However, the tag system provides a much better estimation of clonality when the number of sister cells in each clone exceeds shear site variations. Therefore, clone size was underestimated when considering only shear sites in expanded clones like samples S-3 and S-4. Given this, our tag system should be used for samples with different clonality status to avoid underestimation of the size of clones. See Additional file 2: Figure S3 for a simple comparison of shear site and tag variations.

### Validation of the methodology

**Figure 3:**
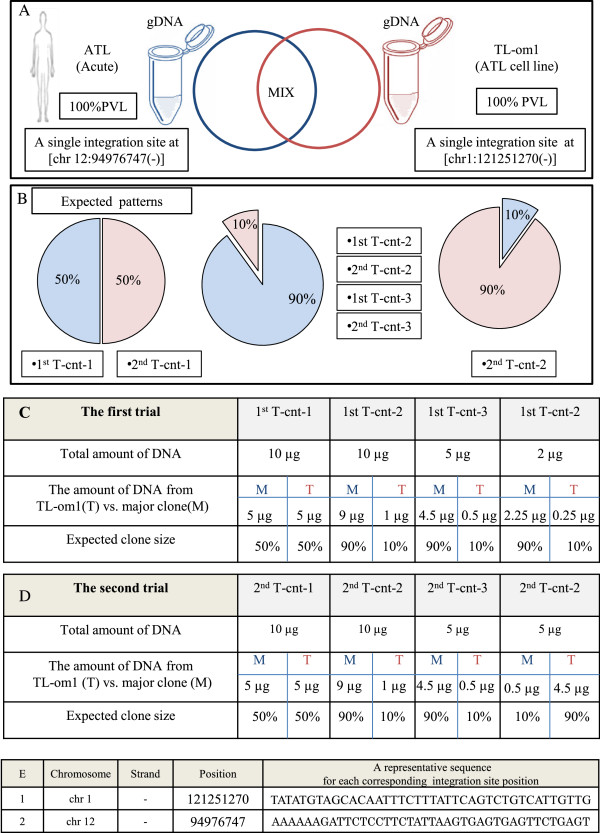
**Preparing the control system. (A)** The control system was designed by mixing sonicated genomic DNA (gDNA) of TL-Om1 with that of an ATL patient in proportions of 50:50 and 90:10. TL-Om1 is a standard ATL cell line with 100% PVL and a known single integration site at (chr1:121251270(-)). The patient sample was from an acute type of ATL with 100% PVL and a single integration site at (chr 12:94976747(-)). **(B)** The expected clonality patterns: (50% *vs.* 50%), (90% *vs.* 10%), and (10% *vs.* 90%) were generated by mixing gDNA from an ATL sample with that from TL-Om1. **(C, D)** Full details of the first trial’s and the second trial’s samples including: name of samples, total amount of DNA (μg), the amount of DNA (μg) from TL-Om1 (T) *vs*. major clone (M), and expected clone size are provided. **(E)** Integration site position of TL-Om1 and the major clone of ATL sample.

Our newly developed method - the tag system and the related data analysis - were successfully validated, internally. As mentioned above, the initial validation was done by analyzing samples from different HTLV-1-infected individuals (Figures 1 and 2). Finally, we conducted a comprehensive internal validation by using an appropriate control with known integration sites and clonality patterns to provide direct evidence for the effectiveness of our system in the clonality analysis. We designed a suitable control because there was not an appropriate control available. Using our system, we could evaluate the method and confirm its accuracy, sensitivity, and reproducibility. We selected two samples with the following special conditions as starting materials for preparing the control system.Sample one (M): DNA from an acute ATL patient with 100% PVLs and a single integration site in the major clone (Figure 3A). The integration site of this sample was first checked with conventional splinkerette PCR, which detected a single major integration site. Subsequently, deep-sequencing data (tags only and combinations) showed that approximately 99% of the PVL accounted for the major clone with an integration site at chromosome 12:94976747(-). A small numbers of clones occupied approximately 1% of the PVL of this sample. Those clones were only detected in the second trial samples for which the external PCR products were not diluted. Therefore, to simplify the overall analysis, we removed those low-abundance clones (data not shown).Sample two (T): DNA was isolated from a fresh culture of TL-Om1, which is a registered monoclonal ATL cell line with 100% PVL and a single integration site at chromosome 1:121251270(-) in each cell (Figure 3A).Having prepared these two samples, they were sonicated and mixed in proportions of 50:50 and 90:10 (Figure 3B). These known proportions were thus expected to generate specific patterns that could be verified with our subsequent analysis. We conducted two independent sets of trials.In the first trial, samples were named as ‘first trial control 1 ~ 4’ and abbreviated as 1st T-cnt-1 ~ 4. Various amounts of DNA (μg) from samples M and T were mixed to prepare the final expected clone sizes as shown in Figure 3C. A 1-μL sample of a 10-fold dilution of external PCR product was used as the starting material for nested PCR for this trial. The samples were run in separate lanes of HiSeq 2000.We named the samples of the second trial as second trial control-1 ~ 4 and abbreviated them as 2nd T-cnt-1 ~ 4. DNA samples were mixed similarly to that for the first trial except for sample four (Figure 3D). In contrast to the first trial, we used 1 μL of the external PCR product without any dilution as a starting material for the nested PCR. These samples were multiplexed and run in the same lane of HiSeq 2000. The purpose of the second trial was to test both method reproducibility and the effect that the dilutions had on the results.

**Figure 4:**
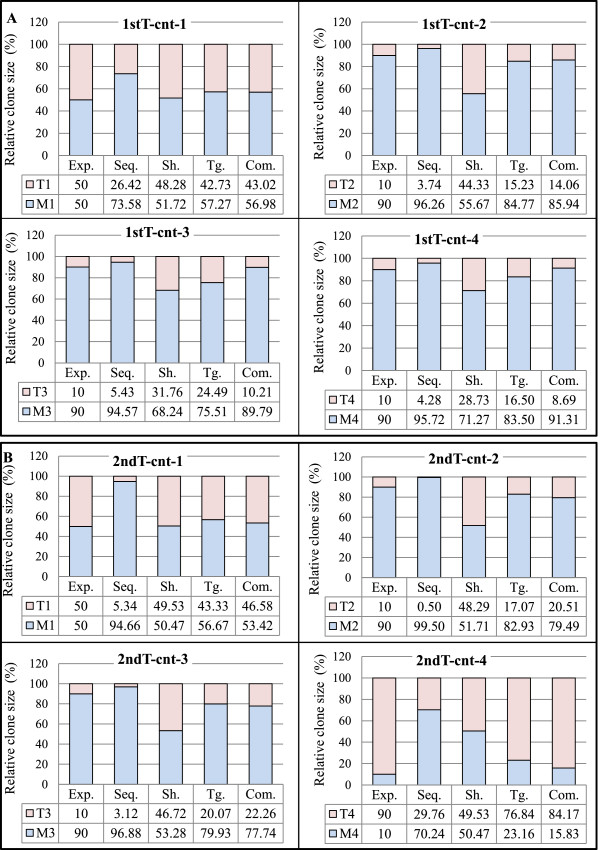
**Validation of the tag system.** For each control sample, both the expected and the experimentally observed patterns of raw sequence reads, shear sites, and the combination of tags and shear sites are represented in the bar graphs. Abbreviations: Com.: Combinations, Exp.: expected pattern, Seq.: raw sequencing data without removing PCR duplicates, Sh.: Shear sites, Tg.: Tags. **(A)** Clone size data of the first trial samples: Data were obtained considering the final optimal conditions: (Bowtie parameters: -v 3 - - best, and filtering condition: (merging approach) JT-10). **(B)** Clone size data of the second trial samples: Data were obtained considering the final optimal conditions: (Bowtie parameters: -v 3 - - best, and filtering condition: (merging approach) JT-10-1%). See Additional file 1: Figure S4 for information on merging approach.

The samples of both the first and second trials were analyzed under the same conditions, except where noted above. For each control sample, expected patterns and experimentally observed patterns were calculated for (a) raw sequence reads, (b) shear sites, (c) only tags, and (d) the combination of tags and shear sites (Figure 4). Figure 4 shows the data when the optimal conditions were considered. Additional file 1: Figure S3 includes most of the data accumulated during optimization of the method.

### Evaluating the accuracy of the clonality analyzed based on shear sites *vs.* tags system

**Figure 5:**
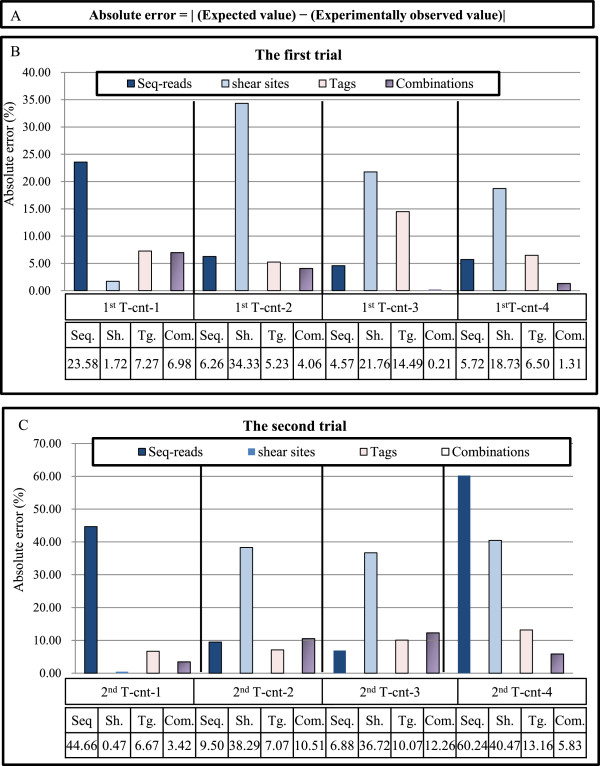
**Evaluating the accuracy of the clonality analysis. (A)** Absolute error is calculated by subtracting the expected values from the experimentally observed values. **(B, C)** The accuracy of the method is evaluated by calculating the absolute error of the clone size estimation of the control samples (see Figure 3). The *y* axis represents the percentage of absolute errors in different conditions including: (1) raw sequencing reads without removing duplicated PCR, (2) only shear sites, (3) only tags, and (4) the combination of tags and shear sites. The absolute errors of the final optimal condition: the first trial: (Bowtie parameters: -v 3 - - best, and filtering condition: (merging approach) JT-10), and the second trial: (Bowtie parameters: -v 3 - - best, and filtering condition: (merging approach) JT-10-1%) are presented in this figure. Please refer to Additional file 1: Figure S6 for the absolute errors in all examined conditions. **(B)** The absolute errors of the first trial. **(C)** The absolute errors of the second trial. See Additional file 1: Figure S4 for information on merging approach.

The ‘absolute error’, a technique used to evaluate system accuracy [61], was used to assess our method. The experimental values were subtracted from expected values (Figure 5A). Taking advantage of our control system (the first and second trial samples), the clone size was calculated by considering (a) sequencing reads without removing PCR duplicates, (b) only shear sites, (c) only tags, and (d) the combination of tags and shear sites (Figure 5B and C). The absolute errors of raw sequence reads for the first trial samples were 23.58, 6.26, 4.57, and 5.72, whereas those of the second trial samples were 44.66, 9.50, 6.88, and 60.24. The magnitude of errors in the first trial was lower than that of the second trial probably due to the dilution of the external PCR products in the first trial. However because dilution reduced the number of covered integration sites, it should be done sparingly and with the purpose of the experiments in mind. The errors when considering only shear sites were 1.72, 34.33, 21.76, and 18.73 for the first trial and 0.47, 38.29, 36.72, and 40.47 for the second trial. Underestimations caused by low shear site variation did not affect the relative size of clones when the expected size of the clones was 50% *vs.* 50%. In this situation, shear sites had the smallest error: 1.72 for 1^st^ T-cnt-1 and 0.47 for 2^nd^ T-cnt-1.

The errors were reduced in the data using the tag system: 7.27, 5.23, 14.49, and 6.50 for the first trial, and 6.67, 7.07, 10.07, and 13.16 for the second trial. In the case of the combination of tags and shear sites, errors were: 6.98, 4.06, 0.21, and 1.31 for the first trial and 3.42, 10.51, 12.26, and 5.83 for the second trial. Interestingly, the samples ‘tags only’ and ‘combinations’ showed similar error levels. Based on these data, our system showed lower absolute errors than when considering only shear sites (Figure 5) (Additional file 1: Figure S4). Owing to differences in analyzed samples and system setups, we could not directly compare our data with published data [22,46]. Indirect evidence, however, provided by shear site analysis of our own data illustrated that our system has lower absolute errors than using the shear site-based methodology.

### *In-silico* analysis

**Figure 6:**
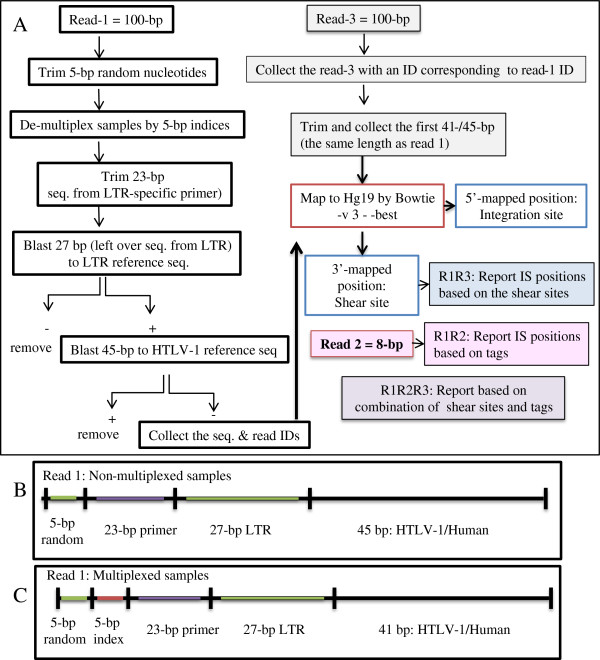
***In-silico *analysis work flow. (A)** Illumina HiSeq 2000 platform outputs raw data of (Read-1 = 100 bp), (Read-3 = 100 bp), and (Read-2 = 8 bp). Data were analyzed according to this work flow after checking quality with the FastQC tool. In the case of Read-1, the first 5 bp were trimmed, and the next 5 bp were used to de-multiplex indexed samples. The downstream 23 bp, which correspond to the LTR primer (F2), were then removed. The next 27 bp were subjected to a blast search against the LTR reference sequence. For the blast search reads, the remaining 41/45 bp were subjected to a blast search against an HTLV-1 reference sequence. Reads were confirmed to be from HTLV-1 was removed, and the sequences and IDs from the remaining reads which considered as human, were collected. Subsequently, Read-3 with IDs corresponding to Read-1’s IDs were collected. The first 41/45 bp of Read-3 were trimmed and collected to have the same length as Read-1. The paired sequences of Read-1 and Read-3 (same lengths) were mapped against hg19 by Bowtie with -v 3 - -best parameters. The 5′-mapped positions were considered to be integration sites and the 3′-mapped positions as shear sites. Read-2 information was used to retrieve the clone size based on tags. Finally, the clone size was computed by combining tag and shear site information. All the analyses were done by our own Perl scripts, which resulted in the following reports. Report R1R3: the distribution of unique shear sites per integration site. Report R1R2: the distribution of unique tags per integration site. Report R1R2R3: the distribution of unique tags and shear sites per integration site. **(B, C)** The structure of Read-1 for the non-multiplexed and multiplexed samples.

Processing, management, and analysis of the large amount of data generated by deep sequencing require special infrastructures and bioinformatics skills. We designed a data analysis and interpretation pipeline specific for HTLV-1 integration sites and clonality studies. The workflow is provided in Figure 6. First, the raw data for high-throughput sequencing were checked for quality by the FastQC tool. We then removed the first 5-bp random nucleotides from read-1 and de-multiplexed those samples that were run in the same lane of the HiSeq 2000 based on 5-bp of the known sequence (Figure 6 and Additional file 1: Figure S2). The downstream 23 nucleotides, which represented LTR-specific primers, were also trimmed before further analysis. We then separated the remaining sequence of read one into two different datasets: (1) LTR sequence and (2) HTLV-1 or human sequence. The former comprises the 27-bp sequence remaining from the LTR, whereas the latter is composed of the 41-bp or 45-bp HTLV-1 or human sequence. In the case of multiplexed and non-multiplexed samples, different lengths (that is, 41-bp and 45-bp) were available for analysis. Both sets were subjected to blast analysis against LTR and HTLV-1 reference sequences with one or two mismatches permitted, respectively. Reads for which the sequence did not match HTLV-1 were presumed to be human as long as their 27-bp LTR sequences matched the LTR reference sequence. The resulting human reads were mapped to the human genome (hg19) using Bowtie 1.0.0 [58]. We employed various parameters of Bowtie and different lengths of read three to obtain the optimal mapping yield (Additional file 1: Table S2). These conditions were achieved when a maximum of three mismatches were permitted (-v parameter) and when the best alignment regarding the number of mismatches was reported (--best parameter). In addition, use of the same length of read-1 as in read-3 allowed for better mapping results. Mapping results are further discussed in Additional file 1: Notes.The 5′-mapped regions were considered to be the positions of integration sites and reported as (chromosome: position: (strand)) for example, (chr1:121251270: (-)). In addition, 3′-mapped regions from read-3 were reported as shear sites for each corresponding position. Information on the tags, obtained from read-2, was used to determine the size of clones as described in subsection: Measuring the size of clones by the tag system. Final outputs of our analysis - the three main reports: R1R3, R1R2, and R1R2R3 - include information on shear sites, tags, and a combination of tags and shear sites, respectively (Figure 6).

### Removing background noise

Data obtained from next-generation sequencers are not error free [40,62-65]. There are many reports on the error rate of Illumina sequencers [66,67]. Teemu Kivioja *et al.* recently developed a system named unique molecular identifiers (UMIs) for quantifying mRNAs and employed filtering criteria to remove false UMIs generated by sequencing errors [68]. In our study, consistent with the data of Kivioja *et al*. [68], the sequencing errors produced false tags with low frequencies. A filtering system was required to remove those tags, which could affect interpretation of our clonality data and reduce the accuracy of the clone size measurement. To minimize the effect of sequencing errors on data interpretation, we tested different filtering conditions to remove background noise. Here, we report our proven filtering approach (Additional file 1: Figure S4).

Considering that tags are designed randomly, each tag has an equal probability of being observed. Hence, the distribution of tags should be fitted to the zero truncated Poisson distribution [59,68]. Therefore, we test data fit to the Poisson distribution to determine the efficacy of each filtering condition. The distribution of tags for each sample was measured by the R-package ‘gamlss.tr’ [59], and the correlation coefficient was compared before and after filtering (Additional file 1: Figure S6).

We used a filtering system, which we named the merging approach. The merging approach was conducted by clustering the tags and allowing only one mismatch so that unique tags, differing only in one nucleotide (one-mismatch permission), were merged. Subsequently, if the frequency of observed tag reads (PCR duplicates) was greater than 10, those unique tags were employed in further analysis. Otherwise, they were considered as artifacts. We referred to this filtering approach as ‘Join Tag- remove10’ (JT-10) in the Figure legends. To facilitate understanding, these filtering conditions are illustrated in Additional file 1: Figure S4.

### Final discussion

The advent of NGS technologies holds promise to reveal the complex nature of neoplasms and to move past the limitations of previous methods. Using different approaches starting from early cytogenetic analysis to later, more elaborate studies with NGS technologies, the clonal composition of different tumors has been analyzed [36-39]. Robust monitoring and tracking of clonal dynamics using provirus integration sites allow for the assessment of clonal composition of HTLV-1-infected individuals from early infection to the final stage of ATL development. To meet the technical requirements for such type of analysis, we combined our expertise in the field of HTLV-1 research and NGS analysis and developed the high-throughput methodology described herein.

Gillet *et al.* also recently introduced a high-throughput method to extensively characterize HTLV-1 integration site preferences and quantify clonality (further discussed in Additional file 1: Notes) [22]. They statistically analyzed shear site data to estimate clone size. According to their published data [22,46] and as well as our current data, the limited variation in shear sites leads to an underestimation of the size of large clones. Considering that the incidence of large clones increases with disease progression from the healthy AC state to the malignant states of smoldering, chronic, or acute [22,46], an accurate measurement of clone size - particularly large clones - is of great clinical significance.

Our study is the first in which the size of large clones was experimentally measured without using statistical estimation. We have provided details of the method design, optimized experiment protocols, and *in-silico* data processing workflow. To validate our methodology and assess its accuracy, we analyzed eight control samples with known integration sites and clone sizes, and four clinical samples. We subjected the samples to deep sequencing so that they had enough read coverage for each integration site and to ensure accurate measurement of clone size (See Additional file 1: Notes). We proved our methodology to be reliable for isolating large numbers of integration sites and to be accurate for quantifying clone size. Because the tag system could provide a sufficient number of variations regardless of clone size, we were able to demonstrate that the measurements are accurate.

Preliminary experiments on the clinical samples with differing PVLs and disease status showed different clonality patterns specific to AC and different ATL disease subtypes. S-1 was selected to represent still-healthy but infected individuals (ACs), S-2 and S-3 to represent indolent types of ATL, and S-4 to represent a typical aggressive type of ATL. Despite similar PVLs, S-1 and S-2 could be distinguished based on clonality patterns (polyclonal *vs.* a shift towards oligoclonal): S-1: AC, 8% PVL, and S-2: SM, 9% PVL. The clones of AC showed a uniform distribution pattern with no large difference in clone size; clones of S-2, however, had non-uniform size. S-2 and S-3 (S-3: SM, 31% PVL) are both smoldering subtypes of ATL progression with differing PVLs (9% *vs.* 31%) and showed similar clonality patterns but a different number of infected cells in each clone. S-3 and S-4 had similar PVL (S-4: acute, 33% PVL) but exhibited different clonality patterns: oligoclonal for S-3 (three or four relatively large clones at the top surrounded with other clones) *vs.* monoclonal for S-4 (a large major clone surrounded with some small clones in the background). After ranking the clones in order of descending size, we noted that the size of the largest clone in the acute sample was 10 times that of the next clone (tags: (chr X: 83705328 (-)) = 2675 *vs.* (chr 14: 30655896 (+)) = 209). Relative size of the major clone (chr X: 83705328 (-)) was also estimated by another method (PCR-southern) (detailed information is provided in Additional file 2: Figure S3 and Additional file 2: Supporting experiments). Samples with distinct disease status (AC, SM, and acute) manifested different clone sizes (Additional file 1: Table S3 and Additional file 2: Table S1 include the number of infected cells in the top 10 clones), but S-1 *vs.* S-2 (0.60 *vs.* 0.67) and S-3 vs. S-4 (0.84 *vs.* 0.80) could not be discriminated based on their oligoclonality index (Additional file 1: Figure S7) (See Additional file 1: Notes for further discussion). Therefore, it can be inferred that, with an accurate measurement of clone size, the application of this method will aid in the discrimination of ATL subtypes. These results suggest a possible association between disease status, PVLs, and clonality patterns. Hence, HTLV-1-infected individuals could be classified in different groups based on their clonality patterns, which could ultimately affect their choice of therapy and estimation of prognosis.

Moreover, by interpreting information from previous studies on HTLV-1 clonality [15,18-20,22,27,31,32,35] and considering the data provided in our present paper, it appears that ACs harbor a polyclonal population of HTLV-1-infected cells, whereas ATL patients show monoclonal patterns. Thus, changes in the clonality pattern and onset of a clonal expansion of HTLV-1-infected cells seem to be potentially applicable as a prognostic indicator of ATL onset. For these purposes, it is necessary to analyze appropriate pools of samples from ACs and different subtypes of ATL and to conduct a cohort study on the clonality patterns of the sequential samples available over time.

## Conclusions

We took advantage of next-generation sequencing technology, a tag system, and an *in-silico* analysis pipeline to develop and internally validate a new high-throughput methodology. The method was proved to accurately measure the size of clones by analyzing control samples with already known clone sizes and clinical samples. We also discussed the novelty, significance, and applications of our method, and compared it with the only existing high-throughput method devised by Gillet *et al.*[22]. Employing our new methodology and the analysis of an appropriate pool of samples provided by JSPFAD [13] will be helpful not only for diagnosis and prediction but also for elaborated understanding of the underlying mechanism of ATL development. The methodology described here could be adapted to investigate and quantify other genome-integrating elements (such as proviruses, transposons, and vectors in gene therapy). In addition, the tag system can be used for quantifying DNA/RNA fragments in RNA expression [68] or in metagenomics for determining the size of bacterial populations.

## Competing interests

The authors declare that they have no competing interests.

## Authors’ contributions

TW, TY, YS, SS, and SF conceived the project. SF designed and carried out the experiments and wrote the manuscript. YL prepared the Perl scripts. YL and SF performed *in-silico* data analysis. SF and TY analyzed and interpret the data. YS, SS and SF contributed in sequencing the samples. YS and KN contributed to *in-silico* data analysis. TY, YL, TW and YS assisted in drafting the manuscript. TY and YS advised the direction of study. TW supervised the study. All authors read and approved the final manuscript.

## Supplementary Material

Additional file 1 **Supplementary data include (1) Supplementary Notes: ‘*Supplementary materials and method*’ and ‘*Supplementary results and discussion*’ (2) Supplementary figures and tables: seven figures, and three tables provided in a PDF file.** Click here for file

Additional file 2 Additional supporting data include (1) Additional supporting protocols and (2) Additional supporting experiments: four figures and one table provided in a PDF file. Click here for file
