# Supplementary material for: Development and validation of a new high-throughput method to investigate the clonality of HTLV-1-infected cells based on provirus integration sites
Source: Genome Med. 2014 Jun 27;6(6):46. doi: 10.1186/gm568 (PMC4097847; doi:10.1186/gm568)
Supplement: Additional file 1 — Supplementary data include (1) Supplementary Notes: ‘ Supplementary materials and method ’ and ‘ Supplementary results and discussion ’ (2) Supplementary figures and tables: seven figures, and three tables provided in a PDF file. [file gm568-S1.pdf]

Additional file 1 - Supplementary data

Development and validation of a new high-throughput method to investigate the clonality of HTLV-1-infected cells based on provirus integration sites

Sanaz Firouzi[1], Yosvany López[2], Yutaka Suzuki[2], Kenta Nakai[3], Sumio Sugano[1], Tadanori Yamochi[1*], Toshiki Watanabe[1*]

[1]Department of Medical Genome Science, Graduate School of Frontier Sciences, The University of Tokyo, 4-6-1 Shirokanedai, Minato-ku, Tokyo 108-8639, Japan

[2]Department of Computational Biology, Graduate School of Frontier Sciences, The University of Tokyo, 5-1-5 Kashiwanoha, Kashiwa-shi, Chiba-ken 277-8561, Japan

[3]Human Genome Center, The Institute of Medical Science, The University of Tokyo, 4-6-1 Shirokanedai, Minato-ku, Tokyo 108-8639, Japan

* Corresponding authors: Tadanori Yamochi[1*], Toshiki Watanabe[1*]

Email addresses:

SF[1]: firouzisanaz1@gmail.com

YL[2]: yosvany@hgc.jp

YS[2]: ysuzuki@k.u-tokyo.ac.jp

KN[3]: knakai@hgc.jp

SS[1]: ssugano@ims.u-tokyo.ac.jp

TY[1]*: yamochi@mgs.k.u-tokyo.ac.jp

TW[1]*: tnabe@k.u-tokyo.ac.jp

Supplementary data

Table of contents

1. Supplementary notes

1.1. Supplementary materials and method

1.1.1. DNA fragmentation

1.1.2. End repair and 5’-phosphorylation

1.1. 3. A-tailing

1.1. 4. Adaptor ligation

1.1.5. Size selection

1.1.6. PCR

1.2. Supplementary results and discussion

1.2.1. Mapping, reads coverage, tag variations, and descriptions related to the clone sizes

1.2.2. Estimating the size of clones by shear sites vs. tags

1.2.3. Oligoclonality index shear sites vs. tag system

2. Supplementary figures and tables

Supplementary Figure S1.

General outline of the high-throughput analysis of HTLV-1 clonality

Supplementary Figure S2.

Outline of the library preparation for sequencing

Supplementary Figure S3.

Measuring clone size using the “merging” filtering approach

Supplementary Figure S4.

Removing background noise

Supplementary Figure S5.

Testing of the data fit to the Poisson distribution

Supplementary Figure S6.

Evaluating the accuracy of clonality analysis for differing conditions

Supplementary Figure S7.

Oligoclonality index for shear sites vs. combinations

Supplementary Table S1.

Required oligonucleotides and reference sequences

Supplementary Table S2.

Samples information and mapping results

Supplementary Table S3.

The top 10 clones isolated from sample S-1, S-2, S-3, and S-4

References

1. Supplementary notes

1.1. Supplementary materials and methods

Wet experiments were performed according the following protocols, and compatible with Illumina. Following Abbreviations were used:

Catalog Number: **#**; Reaction: **rxn**; and Milli-Q water: **MQ**

1.1.1. DNA fragmentation

Shear starting DNA fragments using following equipments and operation settings.

Equipments: Covaris™ S220 System (Applied Biosystems ®) #4465653.

Micro Tube (6×16mm) Round bottom glass tube, AFA fiber with Snap-Cap, Covaris #520045.

Sample: 10 μg DNA in 100 μl MQ. Sonication conditions are similar to Gillet *et al.* [1].

Set the following operation settings for Covaris:

	Cycle 1	Cycle 2
Duty cycle	20%	5%
Intensity	5	3
Cycles per burst	200	200
Time	5 sec	90 sec
Temperature	6-8°C	6-8°C

Check the size of fragments by Agilent 2100 Bioanalyzer Instruments- Agilent DNA 7500 Kit according to the instructions of manufacturer. This product should represent a size range of 300-700 bp (Figure 1B).

1.1.2. End repair and 5’-phosphorylation (for 1 rxn)

End repair converts 3’- and 5’- protruding ends of DNA fragments to blunt ends.

T4 DNA polymerase fills in the 5’overhangs to form blunt ends .It has 5’➔3’polymerase activity and 3’➔5’exonuclease activity, and does not have any 5’➔3’exonuclease function.

Klenow enzyme removes 3’overhangs with a 3’➔5’exonuclease activity. It does not have any 5’➔3’exonuclease activity.

T4 polynucleotide kinase catalyses the transfer of gamma-phosphate from ATP to 5’-OH group of single/double strand DNAs/RNAs. This enzyme phosphorylates the fragments at 5’-end, and makes them ready for ligation reaction.

Set up the following end repair reaction.

Component	μl per tube	Information
Sample	100	The product of Sonication
MQ	43	
T4 DNA polymerase (5U/ μl)	3	Takara (#2040A)
10x DNA polymerase buffer	20	Takara (#2040A)
ATP (10 mM)	20	Takara (#4041)
dNTP mix (25 mM)	8	Invitrogen (#10297-018)
Klenow enzyme (5U/ μl)	1	Takara (#2140A)
T4 polynucleotide kinase (10U/ μl)	5	NEB (#M0201L)
Total	200	Incubate at 20°C for 30 min.

Clean up with PCR purification kit (Qiagen #28104) and elute in 67 μl MQ.

1.1.3. A-tailing (for 1X rxn)

Klenow fragment exo- is the large fragment of DNA polymerase-I with a 5'➔3' polymerase activity without any 3'➔5'exonuclease activity. This enzyme leaves a single base 3'-overhang. Set up the following reaction:

Component	µl per tube	Information
Sample	67	The product of end repair
NEB buffer 2	10	
dATP (1 mM)	20	Takara (#4026)
klenow fragment exo- (15U)	3	NEB (#M0212S)
Total	100	Incubate at 37°C for 30 min

Clean up with Qiaquick PCR purification kit (Qiagen #28104), and elute in 60 µl MQ.

1.1.4. Adaptor ligation

Adaptors were designed compatible with Illumina (Supplementary Figure S2, Additional file 1). Adaptor mixture was prepared as described previously [2]. The sequences of adaptors are provided in Supplementary Table S1. Split the product of A-tailing into 2 tubes of 30 µl then set up the following reaction in a 500 µl thin-layer PCR tube.

Component	µl per tube	Information
Sample	30	The product of A-tailing
Adaptor mix (25 µM)	4	HPLC purified oligonucleotides
T4 DNA ligase buffer (10x)	5	NEB (#M1801)
T4 DNA ligase enzyme (3U/ µl)	5	NEB (#M1801)
MQ	6	
Total	50	Incubate at 20°C for 2 hours

Clean up with MiniElute PCR purification kit (Qiagen #28004), and elute in 20 µl MQ.

1.1.5. Size selection

Size selection is a necessary sample preparation step to remove adaptors carried over from the ligation reaction. Carried over adaptors work as PCR primers, thus interferes the tag data, and lead to a final tag distribution without any sign of PCR amplification (data not shown).

Perform following size selection steps to remove carried over adaptors:

- Prepare a polyacrylamide gel (10% TBE PAGE: 2 mm thick). Pre run 100 volt for 15 min
- Load 100 bp DNA ladder and 20 µl of samples in the wells.
- Run 160 volt for 60 min
- Stain with Ethidium bromide (EtBr) (100 ml 0.5x TBE + 5 µl EtBr) for 10 min.
- Cut the gel from 200-bp to 1000-bp.
- Slice the gel fragment and inset into a 1.5 ml tube which has a pore at the bottom.
- Centrifuge at 12000 rpm for 8 min.
- Add 700 µl of 1x LoTE buffer and centrifuge at 12000 rpm for 2 min.
- Add 1x LoTE buffer up to 2 ml, and Incubate at 65°C for 15 min in a water bath.
- Apply on the Colum (Costar Spin-X centrifuge TUBE Filters 0.22 µm pore CA membrane, #8160).
- Use Millipore Amicon Ultra Centrifugal Filters, 0.5 ml, 100K # UFC 510024 to purify and concentrate (repeat 4 times, each time 500 µl supernatant).
- Wash the filter twice with 500 µl MQ**.**
- End up with purified DNA in 20 µl MQ.

1.1.6. PCR

Although inverse-PCR has been a widely used approach for isolating integration sites, it is relatively inefficient when compared with other PCR-based approaches. It has been reported that splinkerette PCR has become the most widely accepted technique for the amplification of viral and transposon insertion sites [2, 3]. We used a nested-splinkerette PCR to amplify the junction between the human genome and the HTLV-1 insertion (Supplementary Fig. S2). Information on the sequence of primers is available in Supplementary Table S1. F1 is the LTR-specific primer with a sequence complementary to the bottom strand of the target DNA. Because the bottom strand does not have a complimentary region to F1, elongation of the top stand occurs from the 3' hairpin of the adaptor. Therefore, the top strand will not be amplified further. This step reduces the probability of mis-priming. R1 is the adaptor-specific primer with a sequence identical to that of the adaptor. This primer can only undergo amplification until the second cycle of PCR, when the complementary strand is produced by amplification from the F1 primer. Perform external and nested PCR as described below.

External PCR

Component	μl per tube	Information
Template DNA	20	The product of size selection
Primer **F1** (10 μM)	2	
Primer **R1** (10 μM)	2	
10x buffer I	5	
Accuprime taq high fidelity (5U/ μl)	0.2	Invitrogen (#12346-094)
MQ	20.8	
Total	50	

Set the ramping of thermo cycler to 1.9°C /sec.

PCR conditions	Temperature	Time	Cycling
Initial denaturation	94°C	5 min	1 cycle
Denaturation	94°C	50 sec	25 cycles
Combined annealing & extension	68°C	3 min	25 cycles
Final extension	68°C	10 min	1 cycle
Hold @ 4°C			

Mix PCR products of the same sample 50+50+50 µl total: 150µl.

Clean up with Qiaquick PCR purification kit (Qiagen #28104), and elute in 150 µl MQ.

*(optional) Use 1 µl of 10-fold diluted the external PCR for nested PCR.

Nested PCR

Component	µl per tube	Information
DNA	1	*Input from product of external PCR
Primer **P1F2** (10 µM)	2	
Primer **NFCB** (10 µM)	2	
10x buffer I	5	
Accuprime taq high fidelity(5U/ µl)	0.2	Invitrogen (#12346-094)
MQ	39.8	
Total	50	

Set the ramping of thermo cycler to 1.9°C /sec.

PCR conditions	Temperature	Time	Cycling
Initial denaturation	94°C	5 min	1 cycle
Denaturation	94°C	50 sec	30 cycles
Combined annealing & extension	68°C	3 min	30 cycles
Final extension	68°C	10 min	1 cycle
Hold @ 4°C			

Clean up with Qiaquick PCR purification kit (Qiagen #28104), and elute in 50 µl MQ.

Check the size distribution and concentration of PCR products by Agilent 2100 Bioanalyzer Instruments using Agilent DNA 7500 Kit according to the instructions of manufacturer, and conduct sequencing by Illumina HiSeq platform.

1.2. Supplementary results and discussion

1.2.1. Mapping, reads coverage, tag variations, and descriptions related to the clone sizes

As described in Materials and methods, incorporating 5-bp random nucleotides downstream of the region specific for read-1 sequencing primer was necessary to generate high-quality sequencing reads. These 5-bp random nucleotides were not used for S-1, S-2, S-3, and S-4 samples, and thus resulted in a low sequence quality. we handled low quality reads by keeping only the generated sequencing reads that were uniquely mapped, similar to the strategy of Heng Li *et al.* [4]. We utilized different mapping software including Bowtie [5] and Burrows-Wheeler Aligner (BWA). The number of uniquely mapped reads was not significantly different. Bowtie was used for further analysis owing to convenience and higher speed. Final mapped reads were: S-1: 2,758,423; S-2: 281,941; S-3: 4,315,531; and S-4: 11,870,957. Owing to high sequencing quality, the number of uniquely mapped reads was greater for control samples (See Supplementary Table S2.).

Different numbers of generated sequencing reads were analyzed and evaluated. Maximum, minimum, and average mapped reads of our analyzed samples were [1st

T-cnt-1: 27,962,532], [S-2: 281,941], and 10,485,747, respectively. These numbers are comparable to those of published methods in which a maximum, minimum, and average mapped reads of 107509, 4659, and 31961 were used [1].

Depending on the purpose of any particular experiment, a high or a low coverage may be selected. Our data suggest that, although a low coverage (for example S-2: 281,941) can provide some estimation of clonality, a higher coverage may ensure a more reliable and representative picture of clonal composition and isolate a larger number of integration sites. Based on these data, we recommend use of about 2–3 million mapped reads for each analysis. Sequencing and mapping errors are intrinsic to NGS data [4, 6-9]. Therefore, occasional generation of false positive integration sites is unavoidable in this kind of analysis. Considering all the characteristics in NGS data analysis, we designed the study and analysis steps to avoid errors as much as possible, and accurately generate and interpret data.

Tags are randomly generated nucleotides incorporated into splinkerette adaptors (Supplementary Figure S2, and Supplementary Table S1, Additional file 1). Since 7-bp is the default length of read-2 in Illumina, we had initially used 7-bp tags for optimization (S-1, S-2, and S-3). Later it became possible to increase the length of read-2 to 8-bp. Therefore, we analyzed samples with 8-bp tags (S-4, first trial samples, and second trial samples). We also analyzed 2nd trial samples with both 7-bp and 8-bp tags, and compared the results. The measured clone sizes were not significantly different, but barely better in the case of 8-bp tags (data are not shown). Therefore, we used 8 bp as the optimal length for tags in our analysis.

Since, determining a particular number as a general definition for the size of major clone or other clones is too difficult, we employed following definitions. Once the clones are ranked based on their size in a descending order, the following definitions can be applied.

Major clones: High abundant clones at the top of the ranking list.

Minor clones: Low abundant clones at the bottom of the ranking list.

The first major clone: The largest clone at the top

The top 10 clones: The first top 10 largest clones.

1.2.2. Estimating clone size by shear sites vs. tags

Berry *et al.* estimated clone size using shear site data [10]. Considering that there is a nonlinear correlation between shear site variation (fragment lengths) and clone size, they proposed a statistical approach for correcting shear site data. They introduced J, ϕ, and θ as factors referring to possible fragment length, fragment length distribution, and expected number of parent fragments, respectively. J was determined empirically whereas ϕ and θ were statistically calculated. Because we did not use the same experimental setup or statistical estimations, we could not directly compare our data with those of Berry *et al*. Alternatively, the generated variation in shear sites (factor J) might be indirectly compared. Berry *et al.* showed that the relative abundance of small clones was estimated with little bias, but estimating the size of large clones was problematic. For example, they observed a bias greater than 20% when J = 200 and θ was more than 1000. Consistent with their data, we observed underestimations in samples of which the clone sizes exceeded the generable shear site variations. We showed that these underestimations could be overcome by a large variety of tags. In the case of clinical samples (S-1, S-2, S-3, and S-4), maximum generated shear site variations were [S-3: 242] whereas maximum tag variations were [S-4: 2675] (See Supporting Figure 3, Additional file 2).

1.2.3. Oligoclonality index: shear sites vs. tag system

Gillet *et al*. introduced the oligoclonality index (OCI) as a parameter to describe HTLV-1 clonal distribution [1]. OCI was adapted from the Gini coefficient, which has been mainly used in economics to measure income inequality [11]. The ratio of the area between the line of equality (the 45-degree diagonal line) and the Lorenz curve graphically represents the Gini coefficient. Here, we calculated the Gini coefficient using Statsdirect software (http://www.statsdirect.com/), and similar to Gillet *et al*. termed it OCI. Our analysis is based on the data for shear sites, tags, and combinations without any statistical manipulation.

Generally, OCI values range from zero to one. A low OCI indicates a more equal distribution of clones; for example, an OCI of zero represents clones of equal sizes of uniform distribution (perfect equality). A higher OCI indicates a more unequal clone size distribution. An OCI of one represents perfect monoclonality.

The OCI of the samples S-1, S-2, S-3, and S-4 were 0.54, 0.67, 0.68, 0.63, respectively, for shear sites and 0.60, 0.67, 0.84, and 0.80 for combinations. Referring to section of Results and discussion, the clone sizes of S-1 and S-2 measured based on shear site data were similar to those of tags and combinations. Consistent with those data, the OCIs of S-1 and S-2 were similar (shear sites vs. tags vs. combinations: [S-1:0.54 vs. 0.62 vs. 0.60], [S-2: 0.67 vs. 0.68 vs. 0.67]). In the case of S-3 and S-4, however, because the clone size was underestimated by shear sites, the OCI calculated based on shear site data differed from that of tags and combinations (shear sites vs. tags vs. combinations: [S-3:0.67 vs. 0.87 vs. 0.84], [S-4: 0.63 vs. 0.88 vs. 0.80]). Although these samples were categorized based on accurately measured clone sizes, they could not be clearly discriminated based on their OCI. In reference to the limitations of the Gini coefficient addressed in economics, because the Gini coefficient is a relative measure, countries may have identical Gini coefficients even with different income distributions [12]. This problem makes interpretation of the Gini coefficient (and thus OCI) controversial. Therefore, S-3 and S-4, even with different sizes and distributions of clones, had a similar OCI (0.84 vs. 0.80). These data suggest that accurately measured clone sizes are more desirable than OCI for discriminating ATL subtypes.

Supplementary Figure S1.

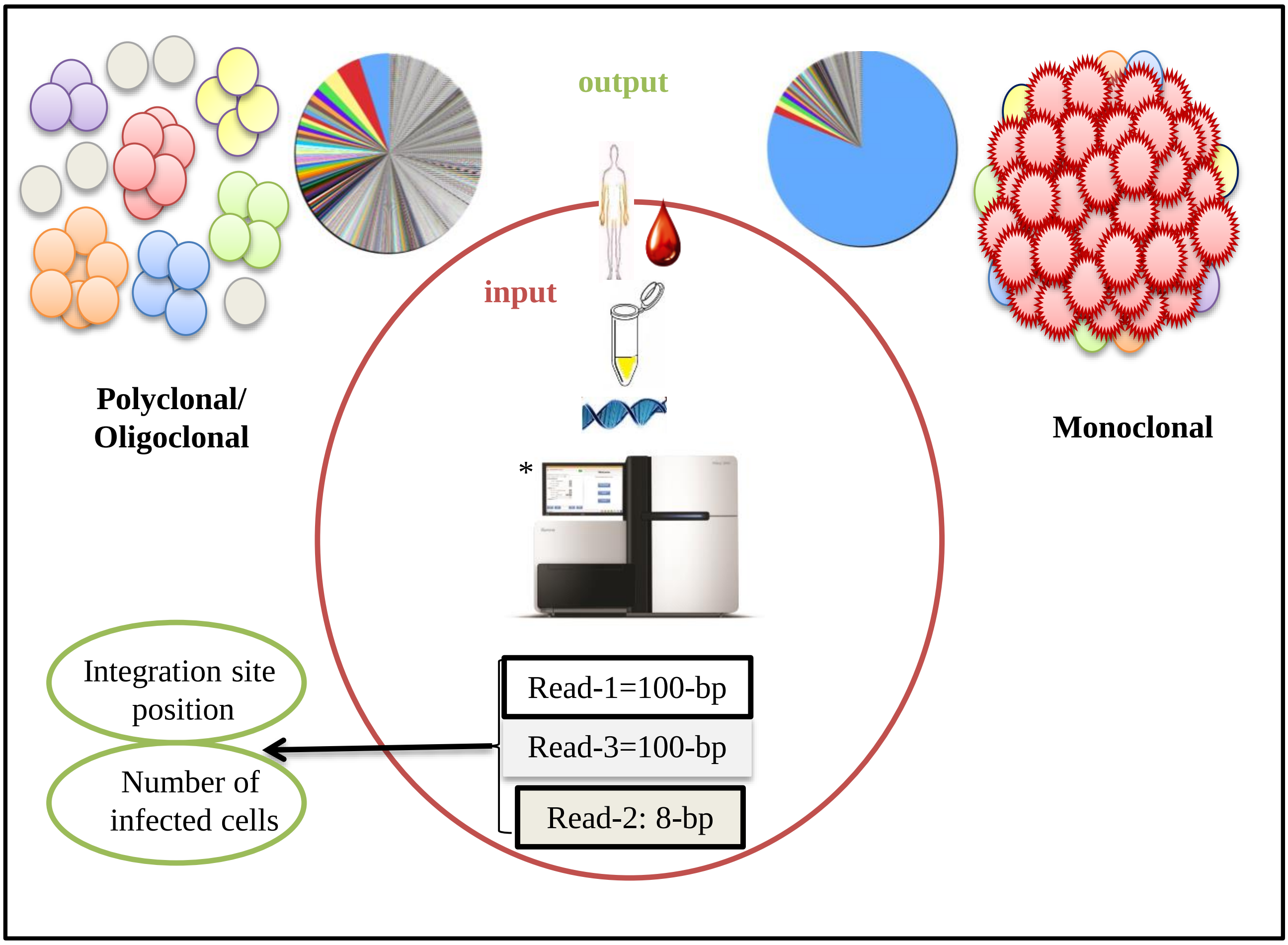

Supplementary Figure S1. General outline of the high-throughput analysis of HTLV-1 clonality Our method uses genomic DNA as the starting material. Data generated by NGS technology undergo bioinformatics analysis using a supercomputer. Processed data provide important information about the position of integration sites and the original number of infected cells in each clone. From this information, we can infer the clonality status of HTLV-1-infected individuals.

* The photo of HiSeq 2000 platform has been used by permission from Illumina (c 2014 Illumina, Inc. All rights reserved.)

Supplementary Figure S2

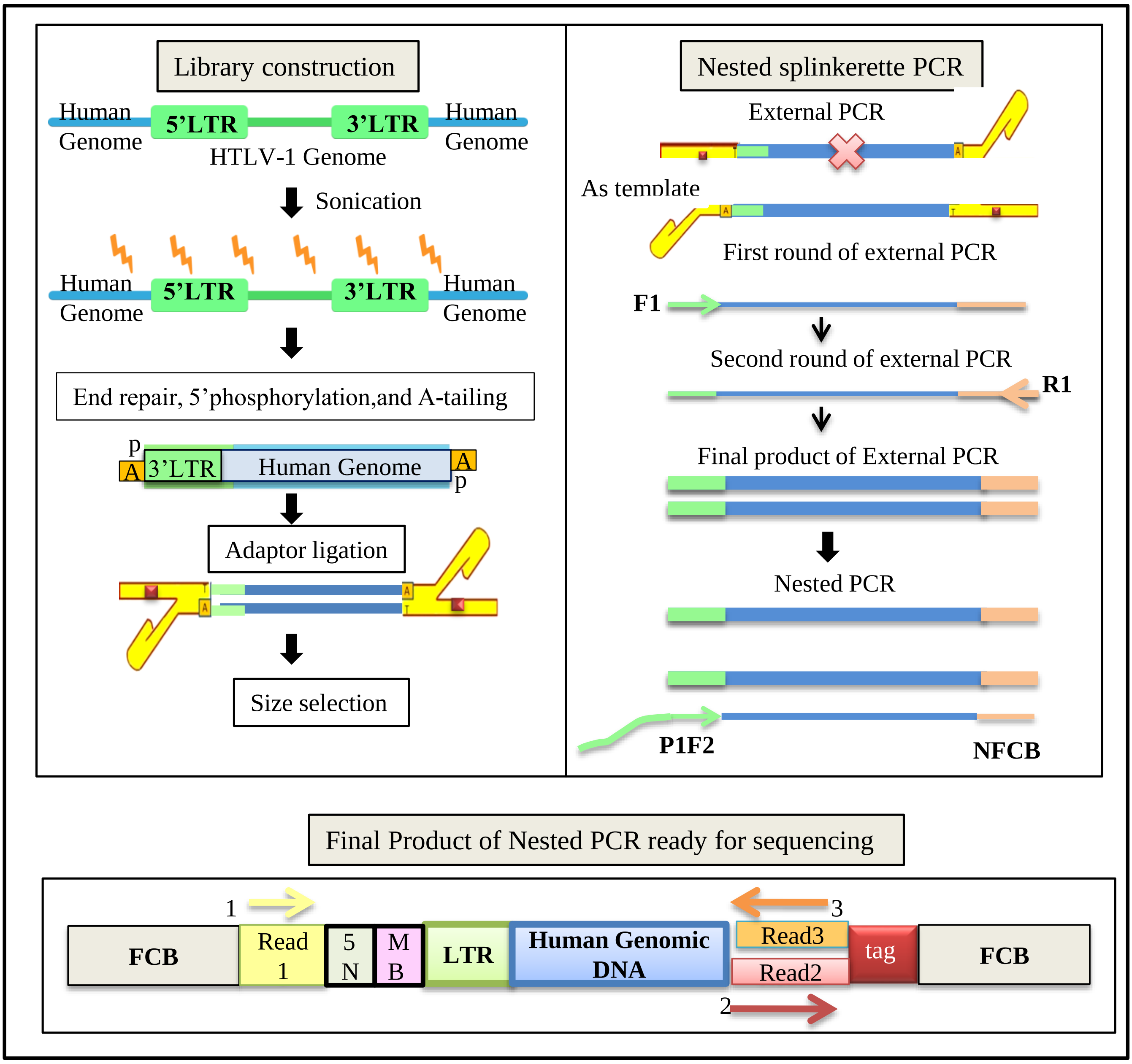

Supplementary Figure S2. Outline of the library preparation for sequencing

(A) Design of primers and the hairpin adaptors leads to specific amplification from integration sites. F1 is the LTR-specific primer with a sequence complementary to the bottom strand of the target DNA. R1 is the adaptor-specific primer with a sequence identical to that of the adaptor. This primer can only undergo amplification until the second cycle of PCR, when the complementary strand is produced by amplification from the F1 primer. After amplification of the target region in external PCR, 1 µl of this product is used as the starting material for nested PCR. Alternatively, the external PCR product is diluted 10-fold, and 1 µl is used for nested PCR.

(B) The final product, ready for sequencing, includes the following regions:

FCB = flow cell binding sequence: 3’-/5’-

Read 1: compatible with the read-1 sequencing primer (5’-read)

Read 2: compatible with the read-2 sequencing primer (8-bp tag read)

Read 3: compatible with the read-3 sequencing primer (3’-read)

5N: 5-bp random nucleotides

MB: 5-bp known multiplexing barcodes including: [barcode 1: ACAGT], [barcode 2: GGCTA], [barcode 3: TTACG], and [barcode 4: GCTAC]

Tag: 8-bp randomly generated nucleotides

Amplified target region: Fragments, amplified from 5’-LTR or 3’-LTR, harbor a portion of HTLV-1 genome or the flanking human genome, respectively. Subsequent *in silico* analysis of sequencing data discriminates the flanking human genome from HTLV-1 genome (see Figure 6).

Supplementary Figure S3.

A

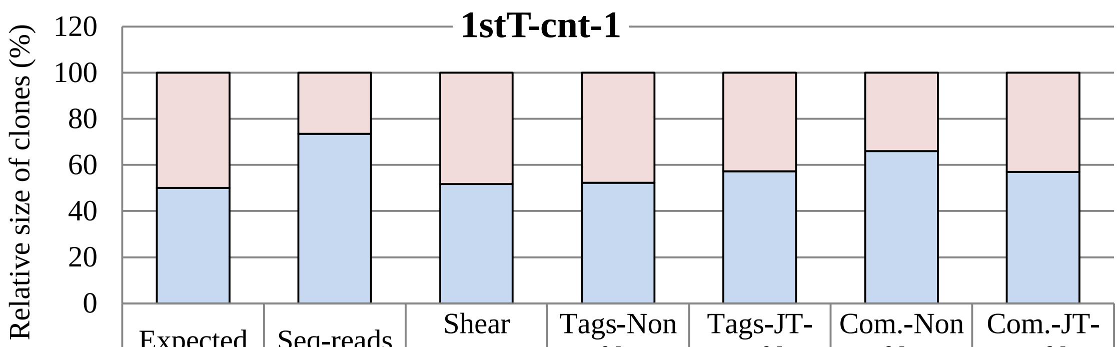

	Expected	Seq-reads	Shear sites	Tags-Non filt.	Tags-JT-10-filt.	Com.-Non filt.	Com.-JT-10-filt.
T1	50	26.42	48.28	47.77	42.73	34.00	43.02
M1	50	73.58	51.72	52.23	57.27	66.00	56.98

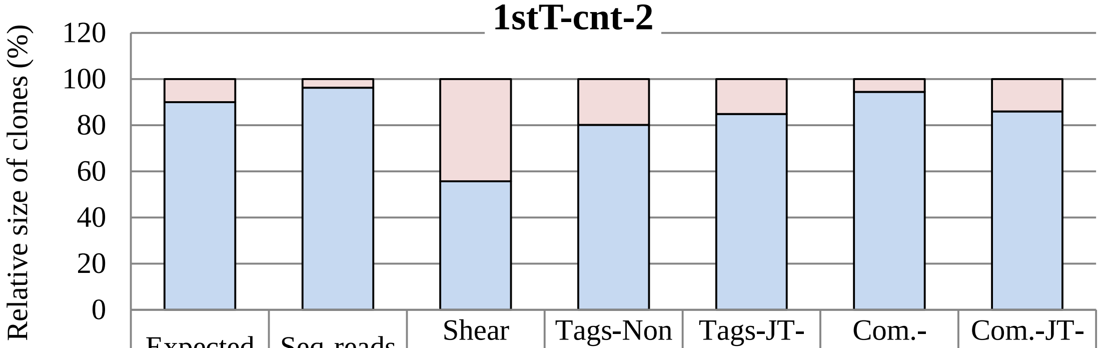

	Expected	Seq-reads	Shear sites	Tags-Non filt.	Tags-JT-10-filt.	Com.-Non filt.	Com.-JT-10-filt.
T2	10	3.74	44.33	19.90	15.23	5.56	14.06
M2	90	96.26	55.67	80.10	84.77	94.44	85.94

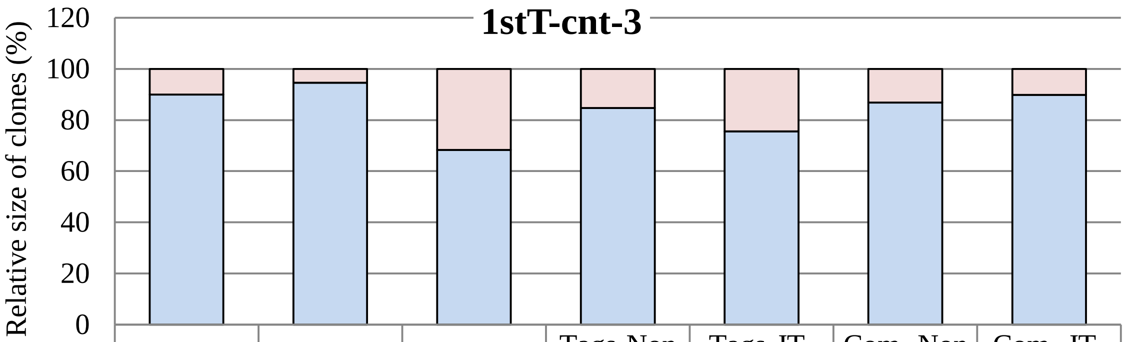

	Expected	Seq-reads	Shear sites	Tags-Non filt.	Tags-JT-10-filt.	Com.-Non filt.	Com.-JT-10-filt.
T3	10	5.43	31.76	15.27	24.49	13.18	10.21
M3	90	94.57	68.24	84.73	75.51	86.82	89.79

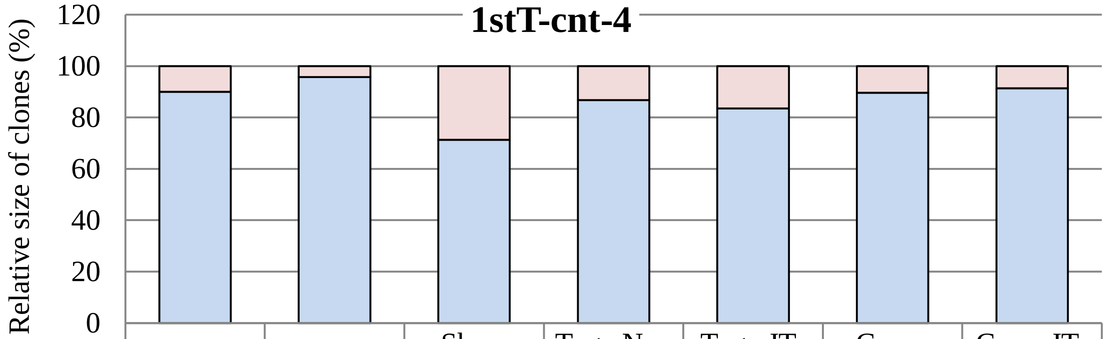

	Expected	Seq-reads	Shear sites	Tags-Non filt.	Tags-JT-10-filt.	Com.-Non filt.	Com.-JT-10-filt.
T4	10	4.28	28.73	13.19	16.50	10.35	8.69
M4	90	95.72	71.27	86.81	83.50	89.65	91.31

Supplementary Figure S3.

B

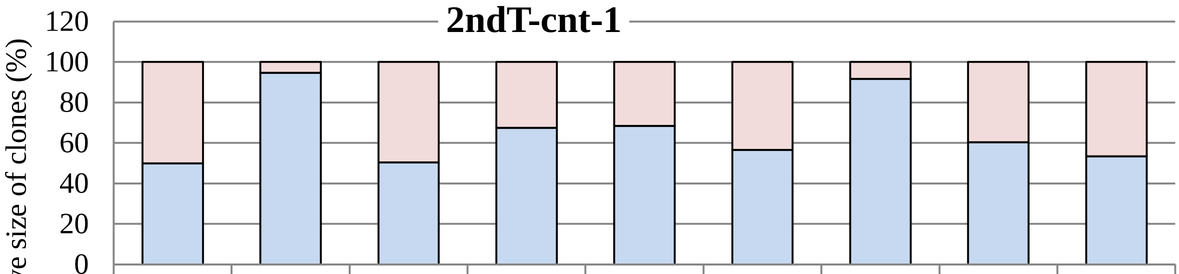

	Expected	Seq-reads	Shear sites	Tags-Non filt.	Tags-JT-10-filt.	Tags-JT-10-1%-filt.	Com.-Non filt.	Com.-JT-10-filt.	Com.-JT-10-1%-filt.
T1	50	5.34	49.53	32.43	31.57	43.33	8.23	39.68	46.58
M1	50	94.66	50.47	67.57	68.43	56.67	91.77	60.32	53.42

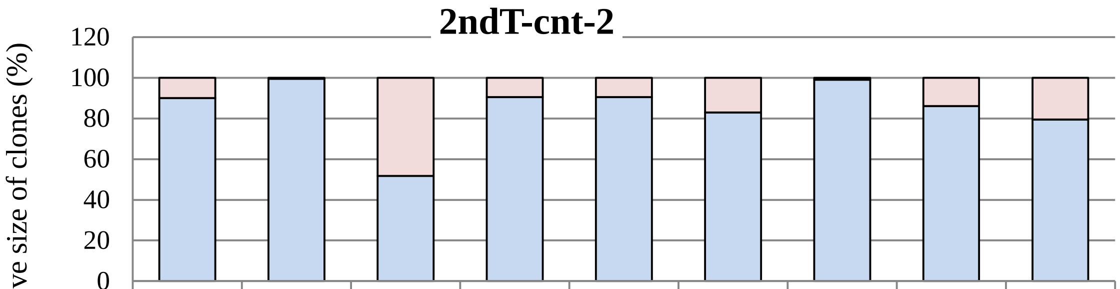

	Expected	Seq-reads	Shear sites	Tags-Non filt.	Tags-JT-10-filt.	Tags-JT-10-1%-filt.	Com.-Non filt.	Com.-JT-10-filt.	Com.-JT-10-1%-filt.
T2	10	0.50	48.29	9.55	9.52	17.07	0.97	13.88	20.51
M2	90	99.50	51.71	90.45	90.48	82.93	99.03	86.12	79.49

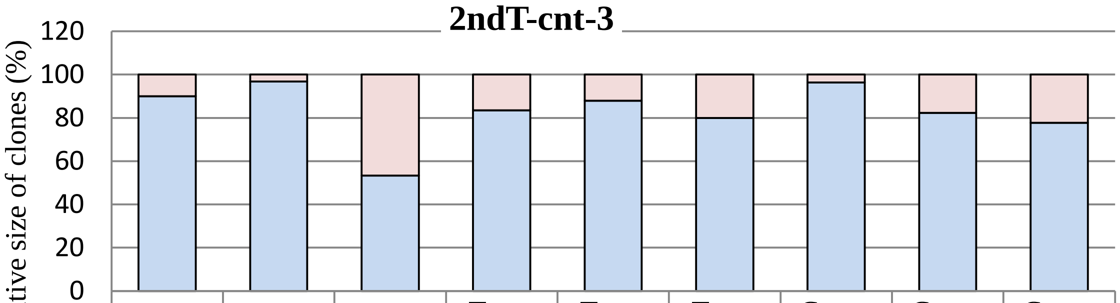

	Expected	Seq-reads	Shear sites	Tags-Non filt.	Tags-JT-10-filt.	Tags-JT-10-1%-filt.	Com.-Non filt.	Com.-JT-10-filt.	Com.-JT-10-1%-filt.
T3	10	3.12	46.72	16.51	12.08	20.07	3.59	17.75	22.26
M3	90	96.88	53.28	83.49	87.92	79.93	96.41	82.25	77.74

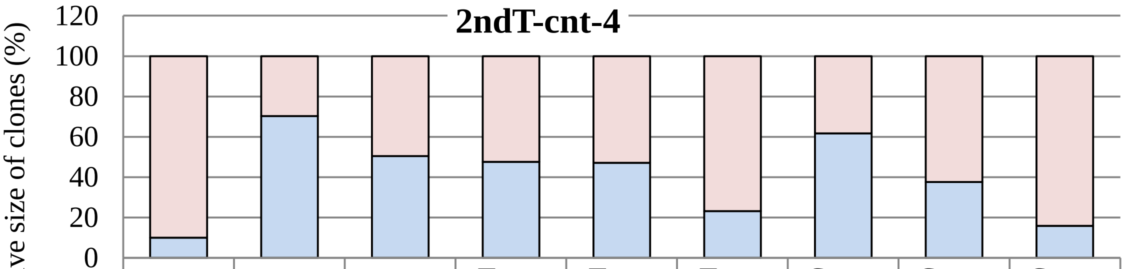

	Expected	Seq-reads	Shear sites	Tags-Non filt.	Tags-JT-10-filt.	Tags-JT-10-1%-filt.	Com.-Non filt.	Com.-JT-10-filt.	Com.-JT-10-1%-filt.
T4	90	29.76	49.53	52.46	52.88	76.84	38.24	62.41	84.17
M4	10	70.24	50.47	47.54	47.12	23.16	61.76	37.59	15.83

Supplementary Figure S3. Measuring clone size using the “merging” filtering approach

The first trial and second trial samples were analyzed as described in Results and discussion and in Fig. 3 and Fig. 4. The size of the clones was based on: (a) raw sequence reads, (b) shear sites only, (c) tags only, and (d) the combination of tags and shear sites.

Data from “raw sequence reads”, and “shear sites only” were not filtered in any of samples. For “tags only” and “the combination of tags and shear sites”, the data are also shown prior to removing background noise (unfiltered). (A) Samples of the first trial. (B) Samples of the second trial. See Fig. 3 for full details of control samples.

Supplementary Figure S4.

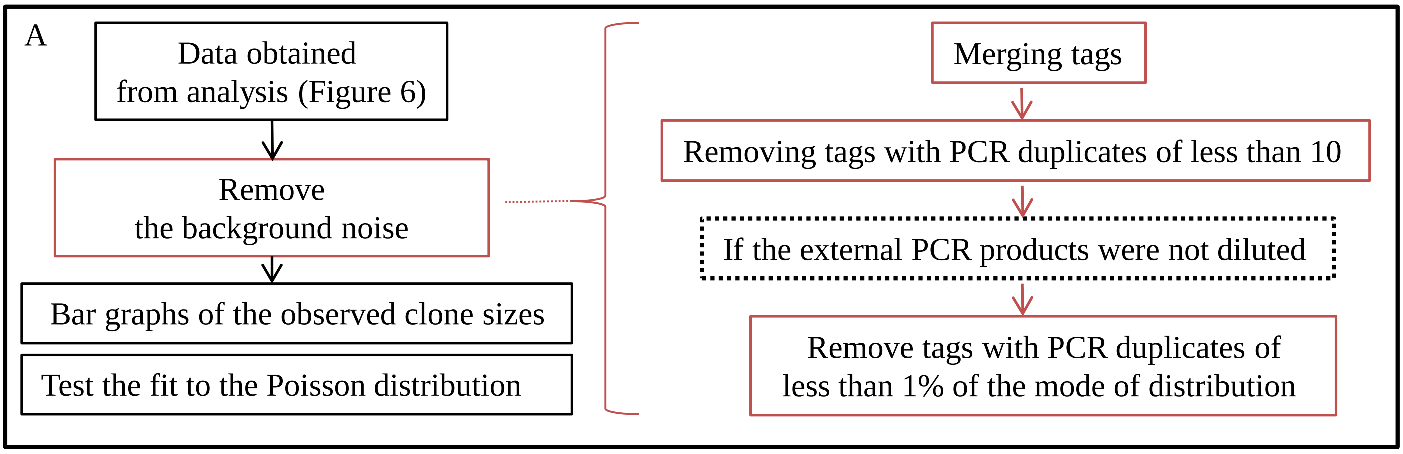

B

	Chromosome	Strand	Position	Tags	PCR duplicates
1	chr 1	-	121251270	ACATGCTA	24
2	chr 1	-	121251270	ACAATCTA	32
3	chr 1	-	121251270	ACATTCTG	22
4	chr 1	-	121251270	ACATACTA	11
5	chr 1	-	121251270	ACATTCTA	28
6	chr 1	-	121251270	CTAGAGGT	39
7	chr 1	-	121251270	CTATAGGT	27
8	chr 1	-	121251270	CAGAAGGC	12
9	chr 1	-	121251270	AAGAACGC	12
10	chr 1	-	121251270	AAGAAGGC	56

Merge the tags with one mismatch

	Chromosome	Strand	Position	Tags	PCR duplicates
1	chr 1	-	121251270	ACATGCTA-ACAATCTA-ACATTCTG-ACATACTA-ACATTCTA	117
2	chr 1	-	121251270	CAGAAGGC-AAGAACGC-AAGAAGGC	80
3	chr 1	-	121251270	CTAGAGGT-CTATAGGT	66

The frequency of unique tags before filtering: **10** ⇨ The frequency of unique tags after filtering: **3**

Supplementary Figure S4.

C

Chromosome	Strand	Position	Shear site	Tag	Frequency
chr12	-	94976747	94976704	CGTGTCGG	**2745**
chr12	-	94976747	94976702	CCACACAC	2598
chr12	-	94976747	94976705	TCAATCCA	2598
chr12	-	94976747	94976701	TAAGCCGC	2569
chr12	-	94976747	94976701	TGACTAGC	2453
chr12	-	94976747	94976704	ATCCCTCG	2421
chr12	-	94976747	94976694	GTTTTCGG	2412
chr12	-	94976747	94976696	GGGGTCCG	2367
chr12	-	94976747	94976680	CCGACACC	2309
chr12	-	94976747	94976674	ACTACGAC	2295
chr12	-	94976747	94976639	CAGTCTGA	1
chr12	-	94976747	94976692	AATGGAAG	1
chr12	-	94976747	94976687	GACACTAC	1
chr12	-	94976747	94976673	AAAGGGTA	1
chr12	-	94976747	94976694	AGCATGGC	1

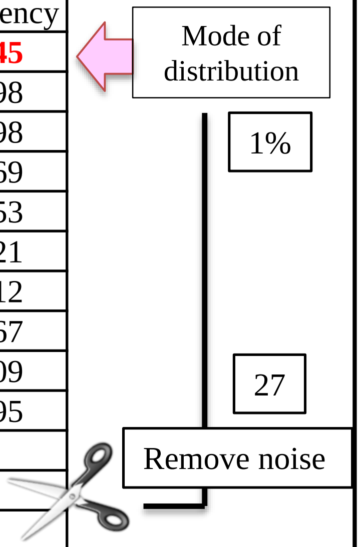

Supplementary Figure S4 . The filtering system for removing background noise
(A) After completing the data analysis as described in Supplementary Figure S3, Additional file 1; we performed a workflow, which included: (1) removing the background noise, (2) fitting data to the Poisson distribution, and (3) preparing graphs of the observed clone sizes. Filtering was done separately for each clone. The background noise was removed by merging tags that differed by one nucleotide (one mismatch permission). Tags with less than ten PCR duplicates were then removed. In the case of the second trial's control samples 1-4 for which the external PCR products were not diluted, tags with PCR duplicates less than 1% of the mode of distribution, were removed (See Supplementary Figure S6, Additional file 1). (B) A simple diagram of merging tags is presented. (C) The external PCR products were not diluted in the second trial (control samples 1-4). For these samples, in addition to the merging approach, tags with PCR duplicates less than 1% of the mode of distribution were removed. Mode of the above depicted distribution is 2745 (indicated in red typeface). In such distribution, tags with a frequency less than 27 (1% of 2745) were removed.

Supplementary Figure S5.

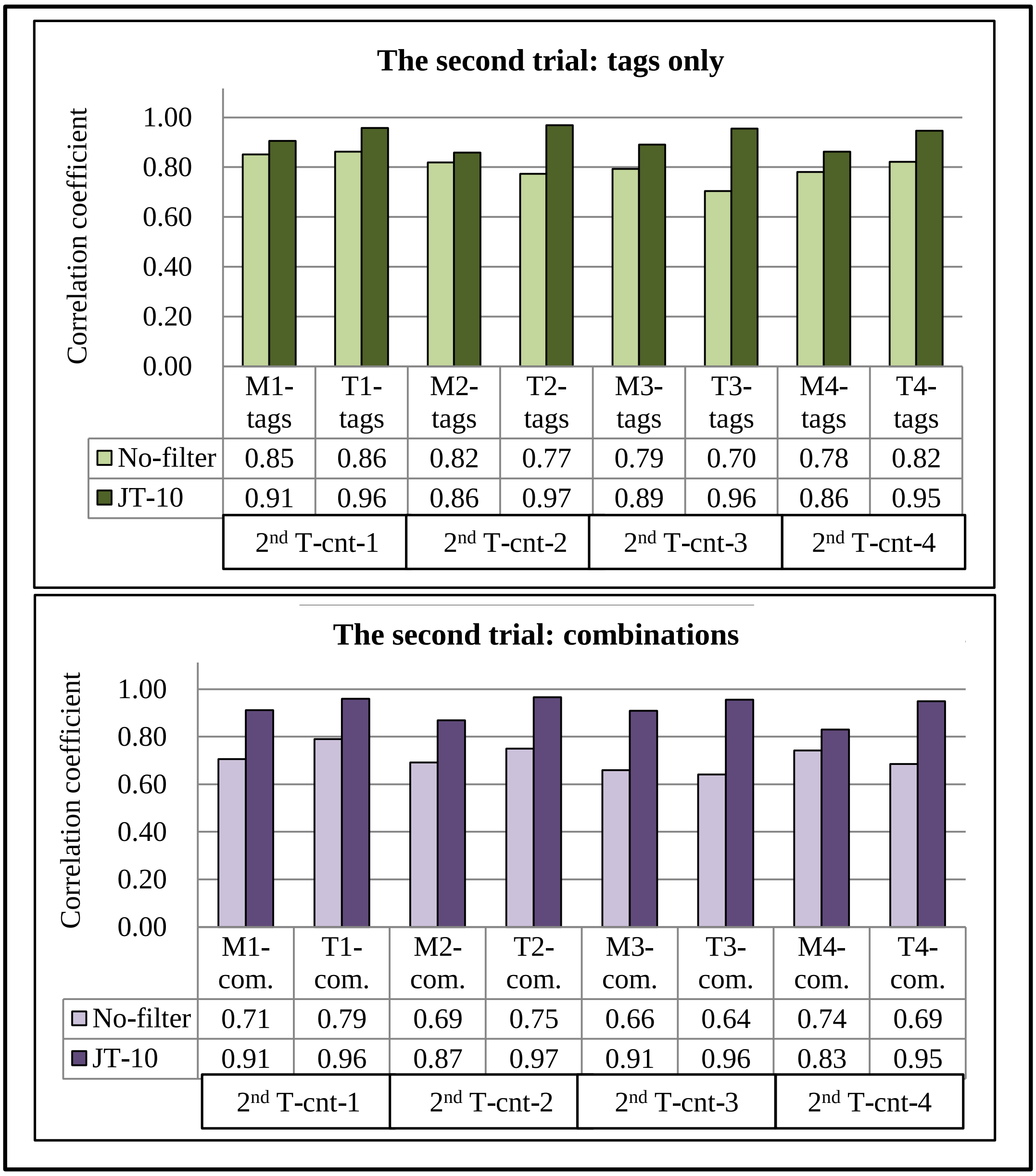

Supplementary Figure S5. Testing of the data fit to the Poisson distribution
The efficacy of filtering was determined by checking the fit to the Poisson distribution. Distribution of tags for each sample was analyzed by R-package “gamlss.tr”, and the correlation coefficient before filtering has been compared to that after filtering.
(A) The second trial: tags only. (B) The second trial: the combination of tags and shear sites.

Supplementary Figure S6.

A

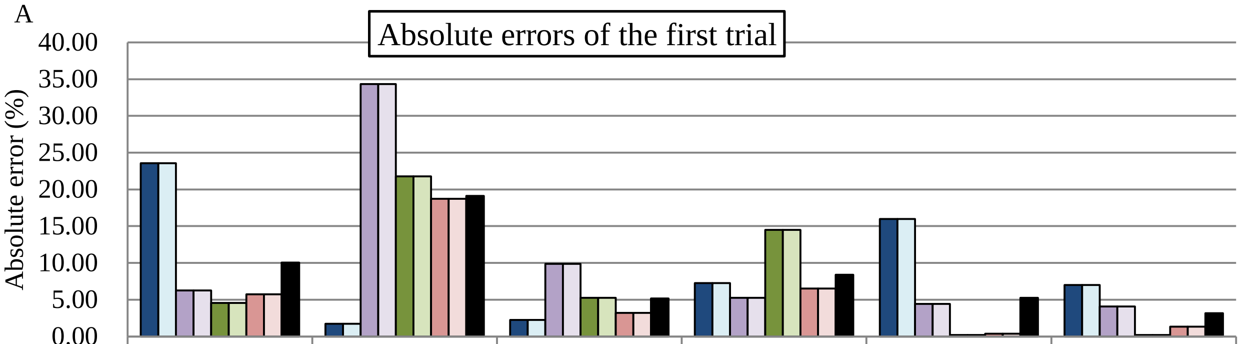

	Seq-reads	shear sites	Tags-Non filt.	Tags-JT-10-filt.	Com.-Non filt.	JT-10-com
M1	23.58	1.72	2.23	7.27	16.00	6.98
T1	23.58	1.72	2.23	7.27	16.00	6.98
M2	6.26	34.33	9.90	5.23	4.44	4.06
T2	6.26	34.33	9.90	5.23	4.44	4.06
M3	4.57	21.76	5.27	14.49	0.21	0.21
T3	4.57	21.76	5.27	14.49	0.21	0.21
M4	5.72	18.73	3.19	6.50	0.35	1.31
T4	5.72	18.73	3.19	6.50	0.35	1.31
average	10.03	19.14	5.15	8.37	5.25	3.14

B

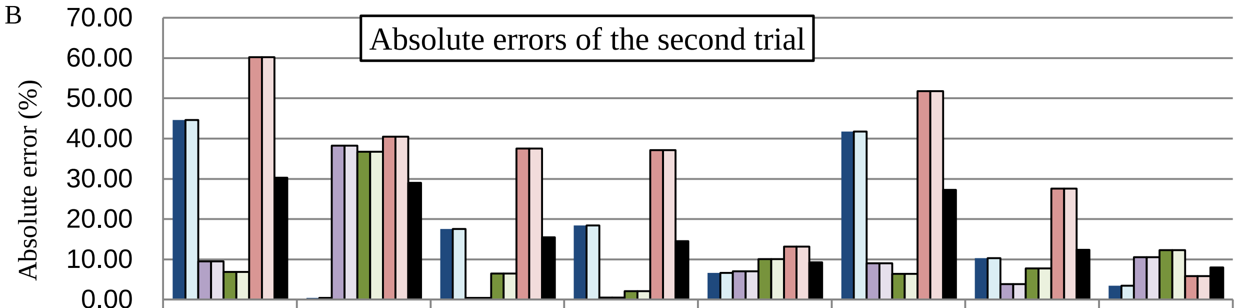

	Seq-reads	shear sites	Tags-Non filt.	Tags-JT-10-filt.	Tags-JT-10-1%-filt.	Com.-Non fil.	Com-JT-10	Com-JT-10-1%
M1	44.66	0.47	17.57	18.43	6.67	41.77	10.32	3.42
T1	44.66	0.47	17.57	18.43	6.67	41.77	10.32	3.42
M2	9.50	38.29	0.45	0.48	7.07	9.03	3.88	10.51
T2	9.50	38.29	0.45	0.48	7.07	9.03	3.88	10.51
M3	6.88	36.72	6.51	2.08	10.07	6.41	7.75	12.26
T3	6.88	36.72	6.51	2.08	10.07	6.41	7.75	12.26
M4	60.24	40.47	37.54	37.12	13.16	51.76	27.59	5.83
T4	60.24	40.47	37.54	37.12	13.16	51.76	27.59	5.83
average	30.32	28.99	15.52	14.53	9.24	27.24	12.39	8.01

Supplementary Figure S6. Evaluating the accuracy of clonality analysis for differing conditions The method was evaluated by calculating "absolute error", which is further described in Results and discussion, Fig. 5, and Fig. 3. Absolute error was calculated for different conditions including: (a) raw sequence reads, (b) shear sites, (c) only tags, and (d) combination of tags and shear sites. In the case of tags only and combinations, both the non-filtered data and the data filtered with the "merging" approach (JT-10 and JT-10-1%) are provided. (A) Absolute errors of the first trial. (B) Absolute errors of the second trial.

Supplementary Figure S7.

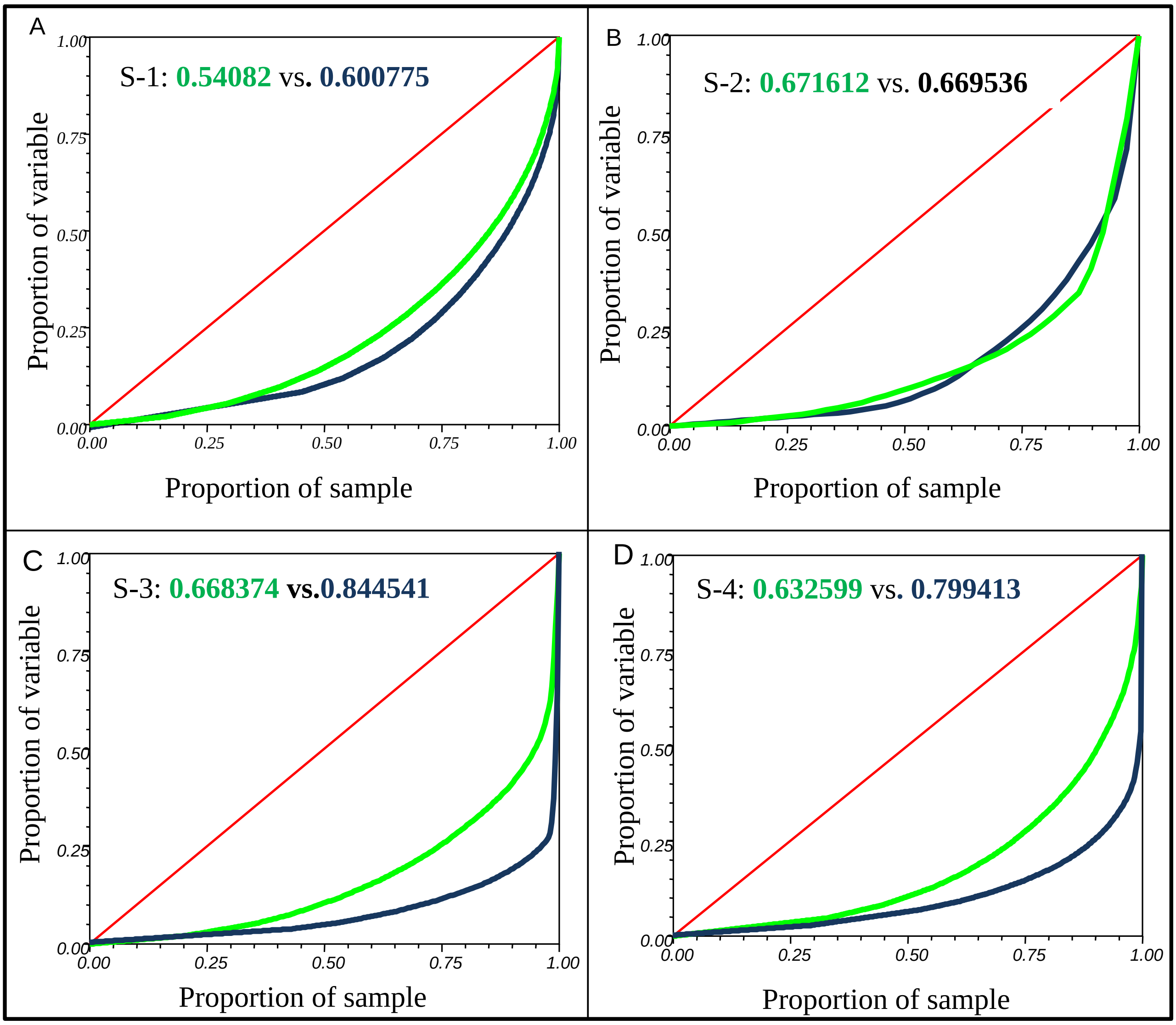

Supplementary Figure S7. Oligoclonality index for shear sites vs. combinations

The Gini coefficient and Lorenz curve were analyzed by StatsDirect software and are represented as an oligoclonality index (OCI). The red, 45-degree diagonal lines are the lines of equality. The green and blue curves are Lorenz curves of shear sites and combination data, respectively. (A) Lorenz curves and the values of OCI for S-1 (shear sites vs. combinations: 0.54082 vs. 0.600775). (B) Lorenz curves and the values of OCI for S-2 (shear sites vs. combinations: S-2:0.671612 vs. 0.669536). (C) Lorenz curves and the values of OCI for S-2 (shear sites vs. combinations: 0.668374 vs. 0.844541). (D) Lorenz curves and the values of OCI for S-2 (shear sites vs. combinations: 0.632599 vs. 0.799413)

Supplementary Table S1. Required oligonucleotides and reference sequences

ID of oligonucleotides	Description	Length	Sequence
Illu-long 8N	Long adaptor strand, includes 8-bp random nucleotides, HPLC purification	86	AACCGTTGCTAGGAGAGACCCAAGCAGA AGACGGCATACGAGATNNNNNNNNGTG ACTGGAGTTCAGACGTGTGCTCTTCCGAT CT
Illu-short-p	Short adaptor stand, 5'- phosphorylated, HPLC purification	27	P-GATCGGAAGAGCGTTTTTTTTCAAAAA
R1	Adaptor primer for External PCR (first PCR)	28	CGAAGAGTAACCGTTGCTAGGAGAGACC
NFCB	Adaptor primer for Nested PCR	21	CAAGCAGAAGACGGCATACGA
F1	LTR-specific primer for External PCR	20	TACCGGCGACTCCGTTGGCT
F2	LTR-specific primer for Nested PCR (second PCR)	23	CCAGCGACAGCCCATCCTATAGC
P1F2 (No-Index)	Includes necessary sequences for Illumina and LTR-specific primer for Nested PCR	81	AATGATACGGCGACCACCGAGATCTACA CTCTTTCCCTACACGACGCTCTTCCGATC TCCAGCGACAGCCCATCCTATAGC
P1F2 (Index1-ACAGT)	Include necessary sequences for Illumina, 5-bp-N, 5-bp- barcode, LTR-specific primer for Nested PCR (F2)	91	AATGATACGGCGACCACCGAGATCTACA CTCTTTCCCTACACGACGCTCTTCCGATC TNNNNNACAGTCCAGCGACAGCCCATCC TATAGC
P1F2 (Index2-GGCTA)		91	AATGATACGGCGACCACCGAGATCTACA CTCTTTCCCTACACGACGCTCTTCCGATC TNNNNNGGCTACCAGCGACAGCCCATCC TATAGC
P1F2 (Index3-TTACG)		91	AATGATACGGCGACCACCGAGATCTACA CTCTTTCCCTACACGACGCTCTTCCGATC TNNNNNTTACGCCAGCGACAGCCCATCC TATAGC
P1F2 (Index4-GCTAC)		91	AATGATACGGCGACCACCGAGATCTACA CTCTTTCCCTACACGACGCTCTTCCGATC TNNNNNGCTACCCAGCGACAGCCCATCC TATAGC
LTR	A portion of HTLV-1 long terminal repeat (GenBank: J02029.1)	27	ACTCTCAGGAGAGAAATTTAGTACACA
HTLV-1	A portion of HTLV-1 genome (GenBank: J02029.1)	50	GTTGGGGGCTCGTCCGGGATACGAGCGC CCCTTTATTCCCTAGGCAATGG

Supplementary Table S2.

Sample	Status	PVL (%)	Barcode	Total reads	Reads from 5'LTR	Reads from 3'LTR	Reads uniquely mapped to Human genome
S-1	AC	7.56	No	101,697,565	37,429,937	64,267,628	2,758,423
S-2	SM	9.01	No	102,690,388	38,344,138	64,346,250	281,941
S-3	SM	31.15	No	96,569,010	31,068,714	65,500,296	4,315,531
S-4	Acute	32.56	No	111,838,665	34,456,523	77,382,142	11,870,957
1st T-cnt-1	TLom1/Acute	100	No	135,665,814	58,159,788	77,506,026	27,962,532
1st T-cnt-2	TLom1/Acute	100	No	108,939,606	46,902,164	62,037,442	22,456,195
1st T-cnt-3	TLom1/Acute	100	No	105,244,134	44,280,981	60,963,153	20,294,502
1st T-cnt-4	TLom1/Acute	100	No	92,804,419	38,245,287	54,559,132	19,736,034
2nd T-cnt-1	TLom1/Acute	100	ACAGT	20,653,487	8,877,796	11,775,691	3,580,966
2nd T-cnt-2	TLom1/Acute	100	GGCTA	31,909,311	13,607,338	18,301,973	5,937,997
2nd T-cnt-3	TLom1/Acute	100	TTACG	15,686,210	6,774,110	8,912,100	2,683,504
2nd T-cnt-4	TLom1/Acute	100	GCTAC	22,110,335	9,443,089	12,667,246	3,950,379

Supplementary Table S2. Sample information and mapping results
Sample information including the disease status, PVL, barcodes, total sequencing reads, and numbers of reads from 5'-LTR, reads from 3'-LTR, and reads uniquely mapped to human genome are presented in this table. Sequencing errors have not included here. The first eight samples were sequenced in separate lanes, and the remaining four samples were barcoded and sequenced in one lane of HiSeq 2000. *In silico* analysis was done by our own Perl scripts, and the sequencing reads were mapped to Hg19 by Bowtie-1 with -v 3 - -best parameters and the same length of read-1 and read-3 were used for mapping. See (Figure 3. Preparing the control system) for more information regarding the last eight samples. PVL of TL-om1 and the control Acute sample were 100% and 100.42%, respectively. In the main manuscript we referred to values of PVLs rounded to zero decimal places: 8%, 9%, 31%, 33%, and 100%. 8-bp random tags were used for all samples except S-1, S-2 and S-3 for which 7-bp tags were used (see Supplementary Notes). Raw sequencing data have been deposited in the Sequence Read Archive with access number of [SRP038906].

Supplementary Table S3. The top 10 clones isolated from sample S-1, S-2, S-3, and S-4. Also see Supporting Table 1, Additional File 2

		Chromosome	Strand	Position	Tags	Relative size (%)	Combinations	Relative size (%)	Shear sites*	Relative size (%)
Top 10 clones of S-1	1	chr11	+	41829319	393	7.08	269	5.30	209	2.60
	2	chr11	-	37042565	329	5.93	235	4.63	130	1.62
	3	chr7	-	121751243	83	1.50	74	1.46	43	0.54
	4	chr13	+	69268469	67	1.21	58	1.14	124	1.54
	5	chr18	-	46701081	65	1.17	58	1.14	30	0.37
	6	chr17	+	18847529	60	1.08	58	1.14	180	2.24
	7	chr15	-	37836845	46	0.83	44	0.87	29	0.36
	8	chr2	-	100184973	44	0.79	42	0.83	22	0.27
	9	chr6	+	10852456	42	0.76	40	0.79	72	0.90
	10	chr8	-	35831701	39	0.70	37	0.73	36	0.45
Top 10 clones of S-2	1	chr15	+	59364370	142	32.27	119	28.95	119	21.25
	2	chr13	-	74706141	55	12.50	52	12.65	11	1.96
	3	chr3	+	28073332	25	5.68	25	6.08	11	1.96
	4	chr21	-	44242161	23	5.23	23	5.60	6	1.07
	5	chr18	-	38428907	19	4.32	19	4.62	1	0.18
	6	chrX	-	107427783	19	4.32	19	4.62	2	0.36
	7	chr13	-	84177236	16	3.64	16	3.89	5	0.89
	8	chr21	+	25834766	15	3.41	15	3.65	7	1.25
	9	chr2	+	234346116	11	2.50	10	2.43	6	1.07
	10	chr7	-	99740574	11	2.50	11	2.68	2	0.36
Top 10 clones of S-3	1	chr4	-	563543	1751	39.76	1192	35.72	242	9.48
	2	chr20	+	58007381	863	19.60	579	17.35	232	9.08
	3	chr5	+	62579369	502	11.40	336	10.07	202	7.91
	4	chr6	+	133958124	210	4.77	207	6.20	191	7.48
	5	chr3	-	126392282	91	2.07	86	2.58	94	3.68
	6	chr3	+	178928610	43	0.98	43	1.29	54	2.11
	7	chr8	+	119096533	27	0.61	27	0.81	43	1.68
	8	chr10	-	111698526	18	0.41	18	0.54	9	0.35
	9	chr13	+	21355493	17	0.39	17	0.51	24	0.94
	10	chr18	+	62126326	16	0.36	14	0.42	10	0.39
Top 10 clones of S-4	1	chrX	-	83705328	2675	51.50	2038	46.54	222	8.35
	2	chr14	+	30655896	209	4.02	160	3.65	87	3.27
	3	chr14	+	49676335	112	2.16	97	2.22	77	2.90
	4	chr6	-	85461536	108	2.08	95	2.17	80	3.01
	5	chr16	-	17339636	102	1.96	97	2.22	98	3.69
	6	chr8	+	96129917	93	1.79	75	1.71	59	2.22
	7	chr1	+	4032445	55	1.06	48	1.10	22	0.83
	8	chr7	+	140001929	50	0.96	49	1.12	40	1.50
	9	chr21	+	35571080	49	0.94	41	0.94	38	1.43
	10	chr1	-	56007274	35	0.67	32	0.73	38	1.43

*Orders and numbers of integration sites in shear sites has been matched to those of tags and combinations.

The reported positions of integration sites can be readily searched by common genome browsers such as Blast of NCBI or Blat of UCSC.

References

1. Gillet NA, Malani N, Melamed A, Gormley N, Carter R, Bentley D, Berry C, Bushman FD, Taylor GP, Bangham CRM: **The host genomic environment of the provirus determines the abundance of HTLV-1–infected T-cell clones.** *Blood* 2011, **117:**3113-3122.
2. Uren AG, Mikkers H, Kool J, van der Weyden L, Lund AH, Wilson CH, Rance R, Jonkers J, van Lohuizen M, Berns A, Adams DJ: **A high-throughput splinkerette-PCR method for the isolation and sequencing of retroviral insertion sites.** *Nature protocols* 2009, **4:**789-798.
3. Devon RS, Porteous DJ, Brookes AJ: **Splinkerettes--improved vectorettes for greater efficiency in PCR walking.** *Nucleic acids research* 1995, **23:**1644-1645.
4. Li H, Ruan J, Durbin R: **Mapping short DNA sequencing reads and calling variants using mapping quality scores.** *Genome Research* 2008, **18:**1851-1858.
5. Langmead B, Trapnell C, Pop M, Salzberg SL: **Ultrafast and memory-efficient alignment of short DNA sequences to the human genome.** *Genome Biol* 2009, **10:**R25.
6. Bravo HC, Irizarry RA: **Model-based quality assessment and base-calling for second-generation sequencing data.** *Biometrics* 2010, **66:**665-674.
7. Nakamura K, Oshima T, Morimoto T, Ikeda S, Yoshikawa H, Shiwa Y, Ishikawa S, Linak MC, Hirai A, Takahashi H, et al: **Sequence-specific error profile of Illumina sequencers.** *Nucleic Acids Res* 2011, **39:**e90.
8. Minoche AE, Dohm JC, Himmelbauer H: **Evaluation of genomic high-throughput sequencing data generated on Illumina HiSeq and Genome Analyzer systems.** *Genome Biol* 2011, **12:**R112
9. Dohm JC, Lottaz C, Borodina T, Himmelbauer H: **Substantial biases in ultra-short read data sets from high-throughput DNA sequencing.** *Nucleic Acids Research* 2008, **36:**e105.
10. Berry CC, Gillet NA, Melamed A, Gormley N, Bangham CRM, Bushman FD: **Estimating abundances of retroviral insertion sites from DNA fragment length data.** *Bioinformatics* 2012, **28:**755-762.
11. Gini C: **Sulla misura della concentrazione e della variabilita dei caratteri.** *Transactions of the Real Istituto Veneto di Scienze* 1914, **LIII**.
12. Maio FGD: **Income inequality measures.** *Journal of Epidemiology and Community Health* 2007, **61:**849-852.
