# Supplementary material for: Development and validation of a new high-throughput method to investigate the clonality of HTLV-1-infected cells based on provirus integration sites
Source: Genome Med. 2014 Jun 27;6(6):46. doi: 10.1186/gm568 (PMC4097847; doi:10.1186/gm568)
Supplement: Additional file 2 — Additional supporting data include (1) Additional supporting protocols and (2) Additional supporting experiments: four figures and one table provided in a PDF file. [file gm568-S2.pdf]

Additional file 2 – Additional supporting data

Development and validation of a new high-throughput method to investigate the clonality of HTLV-1-infected cells based on provirus integration sites

Sanaz Firouzi[1], Yosvany López[2], Yutaka Suzuki[2], Kenta Nakai[3], Sumio Sugano[1], Tadanori Yamochi[1*], Toshiki Watanabe[1*]

[1]Department of Medical Genome Science, Graduate School of Frontier Sciences, The University of Tokyo, 4-6-1 Shirokanedai, Minato-ku, Tokyo 108-8639, Japan

[2]Department of Computational Biology, Graduate School of Frontier Sciences, The University of Tokyo, 5-1-5 Kashiwanoha, Kashiwa-shi, Chiba-ken 277-8561, Japan

[3]Human Genome Center, The Institute of Medical Science, The University of Tokyo, 4-6-1 Shirokanedai, Minato-ku, Tokyo 108-8639, Japan

* Corresponding authors: Tadanori Yamochi[1*], Toshiki Watanabe[1*]

Email addresses:

SF[1]: firouzisanaz1@gmail.com

YL[2]: yosvany@hgc.jp

YS[2]: ysuzuki@k.u-tokyo.ac.jp

KN[3]: knakai@hgc.jp

SS[1]: ssugano@ims.u-tokyo.ac.jp

TY[1]*: yamochi@mgs.k.u-tokyo.ac.jp

TW[1]*: tnabe@k.u-tokyo.ac.jp

Additional supporting data

Table of contents

(1) Additional supporting protocols:
Restriction enzyme digestion, adaptor ligation, external PCR, nested PCR, and southern blotting

(2) Additional supporting experiments

PCR-southern: Results and discussion

Supporting Figure 1: Checking the specificity of technique for isolation of HTLV-1 integration sites by conventional nested- splinkerette PCR using positive and negative controls

Supporting Figure 2: A simple image from an integration site and its shear sites

Supporting Figure 3: A simple comparison of shear site and tag variations

Supporting Figure 4: Detecting the major clone of S-4 by PCR-southern

Supporting Table 1: Information on integration site positions of top-10 clones for each sample

Restriction enzyme digestion

Set up following reaction:

Component	μl per tube	
Genomic DNA	10	2 μg
Sau3A1 enzyme (5U/μl)	4	10 units per μg
10XNEB buffer1.1	4	
MQ	22	
Final volume	40	

Incubate at 37℃overnight (12-16h). Heat inactivate the digested DNA at 65℃for 20 minutes.

Adaptor Ligation

The sequence of adaptors:

Long-strand adaptor (61nt)

CGAAGAGTAACCGTTGCTAGGAGAGACCGTGGCTGAATGAGACTGGTGTCGACACT AGTGG

Short-strand adaptor (48nt)

GATCCCACTAGTGTCGACACCAGTCTCTAATTTTTTTTTTCAAAAAAA

Contains hairpin and GATC 5' overhang

'Long-strand adaptor 'and' Short-strand adaptor' need to be purified by HPLC.

Adaptor ligation reaction

Component	μl per tube	
DNA	6	Sau3AI-digested product (300ng)
Adaptor mix (25 μM)	1	HPLC purified oligonucleotides
T4 DNA-ligase (20 U μl)	0.5	NEB (#M1801)
10× T4 DNA-ligase buffer	4	NEB (#M1801)
MQ	28	
Final volume	40	

Incubate the ligation reaction at 20 °C for 2 hours.

Clean up with PCR purification kit (Qiagen #28104) and elute in 50 μl MQ.

External PCR

Component	μl per tube	Information
Template DNA	20	The product of ligation
Primer **F1** (10 μM)	2	
Primer **R1** (10 μM)	2	
10x buffer I	5	
Accuprime taq high fidelity (5U/ μl)	0.2	Invitrogen (#12346-094)
MQ	20.8	
Total	50	

Set the ramping of thermo cycler to 1.9°C /sec.

PCR conditions	Temperature	Time	Cycling
Initial denaturation	94°C	5 min	1 cycle
Denaturation	94°C	50 sec	30 cycles
Combined annealing & extension	68°C	3 min	30 cycles
Final extension	68°C	10 min	1 cycle
Hold @ 4°C			

Clean up with Qiaquick PCR purification kit (Qiagen #28104), and elute in 50 μl MQ.

*(optional) Use 1 μl of 10-fold diluted the external PCR for nested PCR.

Nested PCR

Component	μl per tube	Information
DNA	1	*Input from product of external PCR
Primer **F2** (10 μM)	2	
Primer **R2** (10 μM)	2	
10x buffer I	5	
Accuprime taq high fidelity(5U/ μl)	0.2	Invitrogen (#12346-094)
MQ	39.8	
Total	50	

Set the ramping of thermo cycler to 1.9°C /sec.

PCR conditions	Temperature	Time	Cycling
Initial denaturation	94°C	5 min	1 cycle
Denaturation	94°C	50 sec	30 cycles
Combined annealing & extension	68°C	3 min	30 cycles
Final extension	68°C	10 min	1 cycle
Hold @ 4°C			

PCR-southern

We conducted a PCR-southern as described followings.

Sample preparation:

Digest 2μg of gDNA by Sau3AI restriction enzyme according to aforementioned protocol.

Perform adaptor ligation as described above. Amplify the ligation product by 30 cycles of an external PCR using LTR-specific (F1) and adaptor-specific (R1) primers.

F1: TACCGGCGACTCCGTTGGCT

R1: CGAAGAGTAACCGTTGCTAGGAGAGACC

Electrophorese the PCR products on a 3% TAE agarose gel, and then transfer on a nylon membrane (Biodyne® Nylon Transfer Membranes (B) of Pall cooperation) for 6 hours. Wash the membrane by 2x Saline Sodium Citrate (SSC) buffer on a shaker at room temperature for 10 min.

Prepare following probes, and then label them using TaKaRa *Bca*BEST Labeling Kit (cat No. 6046) according to the manufacturer's instructions.

[α-32P] dCTP, 0.250 mCi (NEG-513H) was purchased from Perkin Elmer.

LTR-specific (75-bp)

TGTGTACTAAATTTCTCTCCTGAGAGTGCTATAGGATGGGCTGTCGCTGGCTCCGAGC
CAACGGAGTCGCCGGTA

Blue clone-specific (chr-x) probe (171-bp)

GGTGAGATTGCTTTCTTGTAGGCAGTATATAGTGGAGTGATGGTTTTTTTTGTTGTTGT
CCATTTAGCCAGTCTATATATTTTAAGTGGAAAGTTTAATTCATTTATATTCAAAATCAT
AATTGATATGTGAATATTTATTCCTGTCATTTTACTAGTTGATTTCTGGTGG

Red clone-specific (chr-14) probe (196-bp)

GCTCACAGTATTAGAGTGGGTTACATTTTAAGTAGAAAAACATTTGGTTATATCATTGT
CCTTATAGCATGATTCTGACTTATTTGCATAAACAAATATTTATGTTCTTGTTTATGTATT
TTTGTAAAACAATATCTATAGGAAAAGTAGGCCTATCCTATAAACCCCCGGAAGGGAA
GGTTGATTCAGACACAGT

Pre-hybridize the membrane at 65°C for 12 hours and hybridize it at 65°C for 12 hours (on a rotator).

Use 2x10^6 cpm of the labeled probes for hybridization.

Wash the hybridized membrane as followings.

All buffers must contain 0.1% SDS.

2x SSC: at 65°C, 10 min, repeat 3 times

0.5x SSC: at 65°C, 10 min, repeat 3 times

0.2x SSC: at 65 °C, on a shaker water bath for 30 min.

0.1x SSC: at 65°C, on a shaker water bath for 30 min.

Expose the membrane on Carestream Health X-OMAT AR (XAR) Autoradiography Film (KODAK 1651454) for about 3-4 hours. Process the exposed film using FPM 800A, Fuji Film instrument.

(2) Additional supporting experiments

PCR-southern: Results and discussion

We considered PCR-southern as the most appropriate classical approach with less bias compared to other conventional methods, therefore we selected it as the method to provide clear data about the relative size of the major clone in sample S-4. Since HTLV-1-infected clones contain the same LTR sequence, they are detectable using a common LTR probe by PCR-southern (Experiments were performed as described in Additional supporting protocols, Additional file 2). We used LTR specific probe to detect the clones. Size of clones was estimated by the strengths of bands detected from each specific clone. We then compared the relative size of clones measured by PCR-southern with that of Shear site or Tags system. Relative size of clones based on PCR-southern data was so similar to that of our tag system. Together, we successfully could examine the accuracy of our tag system by another method.

Following is in detail information obtained from PCR-southern experiments (Also see Supporting Figure 4).

Two bands detected from S-4 were sequenced. The upper band: chr X: 83705328 (-): from the major clone (The Blue clone).

The bottom band: amplification from 5' LTR containing a part of HTLV-1 genome.

No bands were detected from the Red clone (the second clone of S-4: chr 14: 30655896(+).

We compared the quantitative size of clones measured by shear site and tag system with the qualitative estimation of clone size based on PCR-southern data.

The shear sites data showed that the Blue clone was only 2.6 times larger than the Red clone. If the size estimation by shear sites (blue vs. red: 222 vs. 87) was accurate, in the case that a strong band was detected from the Blue clone, at least a weak band should be detected from the red clone. However, PCR-southern detected a strong monoclonal band from the blue clone, and no band from the Red clone. The data are consistent with the tag data based on which the size of the blue clone is 12.8 times larger than the Red clone (blue vs. red: 2675 vs. 209).

We compared clone size data obtained from S-4 with those of S-1 by considering the information on size of the first clone, and the number of integration sites. S-1 was used as a clone size control. Consistent with shear site and tag data, PCR-southern did not detect any major band from S-1. The number of integration sites isolated from each sample (S-1: and S-4) were1030 and 384, respectively.

Based on the shear site data, the size of first clone in the sample S-1 was 209; and that of S-4 was 222 (similar sizes). However, based on the tag only data, the size of first clone in the sample S-1 was 393; and that of S-4 was 2675 (different sizes). If the size estimation of the

first clone of S-4 (222) was similar to that of S-1 (209) (based on the shear site data), the sample S-4 should show a smear like pattern similar to that of S-1(based on the PCR-southern data). However, the data of PCR-southern (detecting a monoclonal band from S-4) were consistent with those of tag system based on which the the size of first clone in the sample S-4 (S-1 vs. S-4: 393 vs. 2675) was significantly different from S-1.

Taken together the data further support the accuracy of the clone size measured by the tag system, and suggest underestimation of the size of large clones by the shear sites.

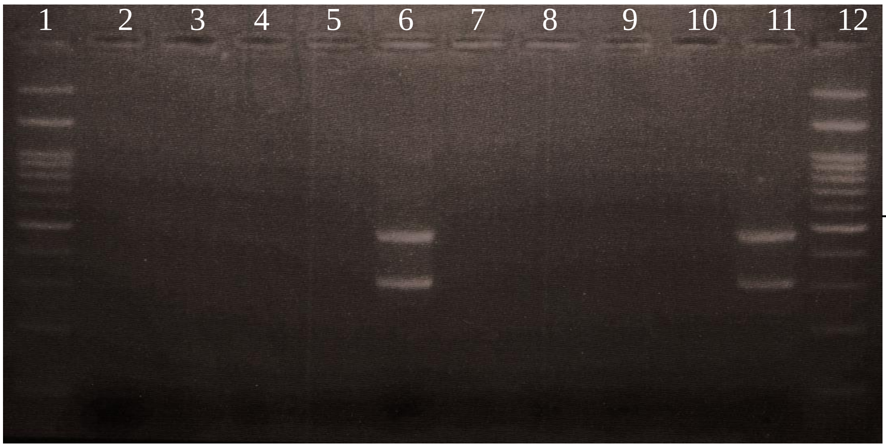

5µl template DNA	1.	100-bp DNA ladder
	2.	U1
	3.	OM-10
	4.	ACH2
	5.	Normal PBMC
	6.	TL-Om1
10µl template DNA	7.	U1
	8.	OM-10
	9.	ACH2
	10.	Normal PBMC
	11.	TL-Om1
	12.	100-bp DNA ladder

Supporting Figure 1.
Checking the specificity of technique for isolation of HTLV-1 integration sites by conventional nested-splinkerette PCR using positive and negative controls
The specificity of method was examined by conventional nested-splinkerette PCR (See Additional supporting protocols for detailed techniques).We analyzed 3 cell lines from HIV (U1, OM10.1 and ACH-2) as a control for exogenous retroviruses. Neither bands nor any smear-like pattern were detected in any of them. gDNA of normal PBMC and TL-Om1 were used as negative and positive controls respectively.

Supporting Figure 2

A simple image from an integration site and its shear sites (Also see Figure 1 and 6)

Read-1 starts with LTR sequence of HTLV-1: “CCAGCGACAGCCCATCCTATAGCACTCTCAGGAGAGAAATTTAGTACACA”.

This region follows with a sequence which its position can be determined by mapping to the human genome. The starting position of human flanking region is defined as integration site (marked by a red asterisk*) .

Read-3 contains fragments with different lengths which were generated by sonication. Mapping results of the fragments with different lengths provides positions of shear sites (marked by a dolor sign $).

*CCTTTATAGGTGAGATTGCTTTCTTGTAGGCAGTATATAGTGGAGTGATGGTTTTTTTTGTTGTTGTCCATTTAGCCAGTCTATATATTTTAAGTGGAAAGTTTAATTCATTTATATTCAA$

*CCTTTATAGGTGAGATTGCTTTCTTGTAGGCAGTATATAGTGGAGTGATGGTTTTTTTTGTTGTTGTCCATTTAGCCAGTCTATATATTTTAAGTGGAAAGTTTAATTC$

*CCTTTATAGGTGAGATTGCTTTCTTGTAGGCAGTATATAGTGGAGTGATGGTTTTTTTTGTTGTTGTCCATTTAGCCAGTCTATATATTTTAAGTGGAAA $

*CCTTTATAGGTGAGATTGCTTTCTTGTAGGCAGTATATAGTGGAGTGATGGTTTTTTTTGTTGTTGTCCATTTAGCCAGTCTATATATTTTAAGTGG$

*CCTTTATAGGTGAGATTGCTTTCTTGTAGGCAGTATATAGTGGAGTGATGGTTTTTTTTGTTGTTGTCCATTTAGCCAGTCTATATATTTTA$

*CCTTTATAGGTGAGATTGCTTTCTTGTAGGCAGTATATAGTGGAGTGATGGTTTTTTTTGTTGTTGTCCATTTAGCCAGTCTATATATT$

*CCTTTATAGGTGAGATTGCTTTCTTGTAGGCAGTATATAGTGGAGTGATGGTTTTTTTTGTTGTTGTCCA$

*CCTTTATAGGTGAGATTGCTTTCTTGTAGGCAGTATATAGTGGAGTG$

*CCTTTATAGGTGAGATTGCTTTCTTGTAGGCAGTATATAGT$

*CCTTTATAGGTGAGATTGCTTTCTTGTAGGCAGTATAT$

*Integration site
$ Shear site

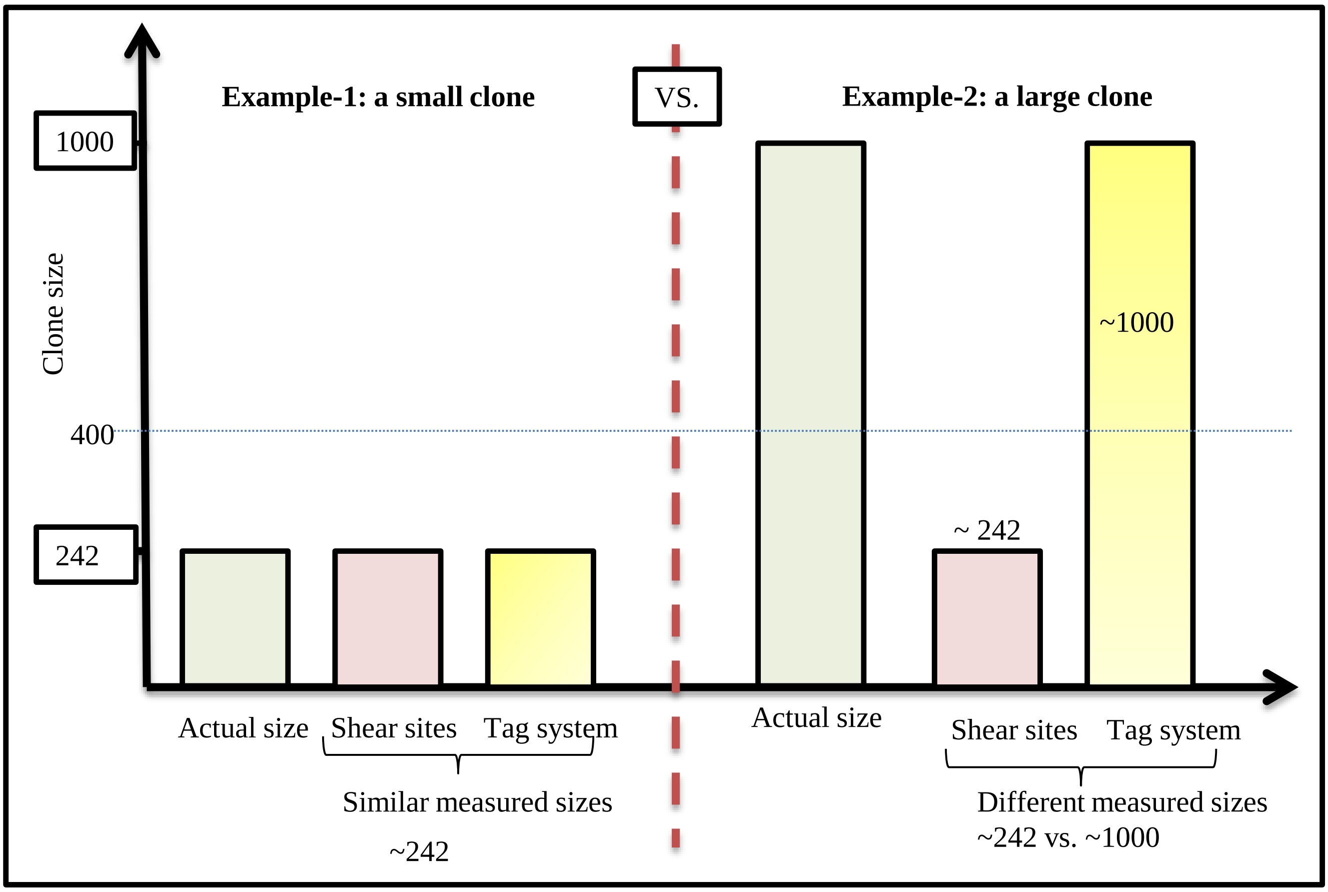

Supporting Figure 3.
A simple comparison of shear site and tag variations
A simple image representing that the two approaches result in the similar clone sizes when the size of clones are small enough to be covered by shear site approach (Example-1).
However, when the size of clones exceeds the shear site variations (242 based on our experiments), clone sizes get underestimated by the shear site approach. Thus, the size of large clones are only measureable by the tag system (Example-2). 400 is the theoretical upper-limit of shear site variations.

Supporting Figure 3.

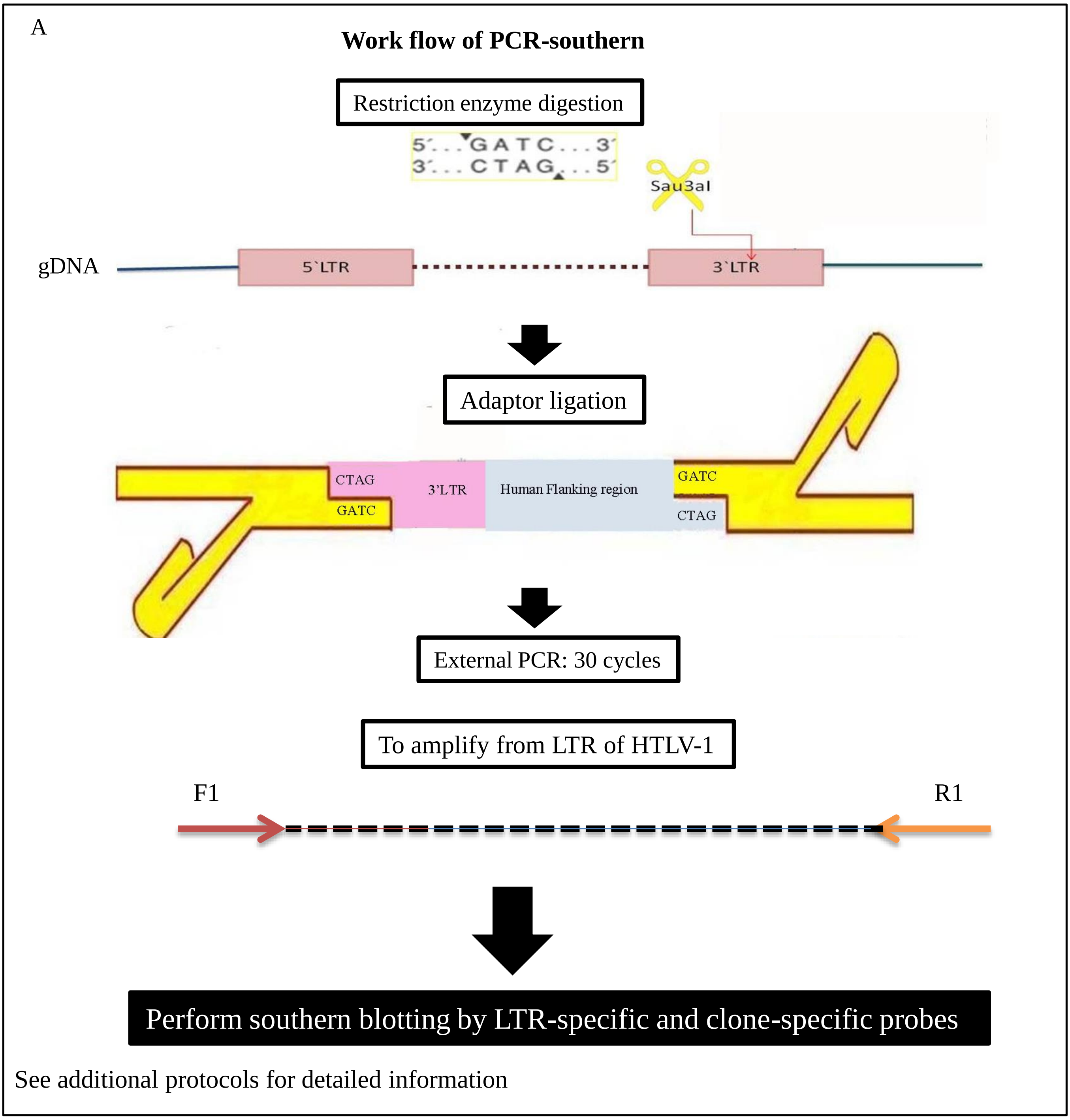

Supporting Figure 3. Detecting the major clone of S-4 by PCR-southern

Work flow of PCR-southern: restriction enzyme digestion, adaptor ligation, external PCR amplification and southern blotting. Perform experiments as described in additional protocols. (B) clones detected by a LTR-specific probe (C) chrx:83705328(-) clone detected by the clone-specific probe (D) Comparison of the clone size estimated by shear sites and the tag system with that of PCR-southern.

Supporting Figure 3. Detecting the major clone of S-4 by PCR-southern

B

Detected by LTR-specific probe

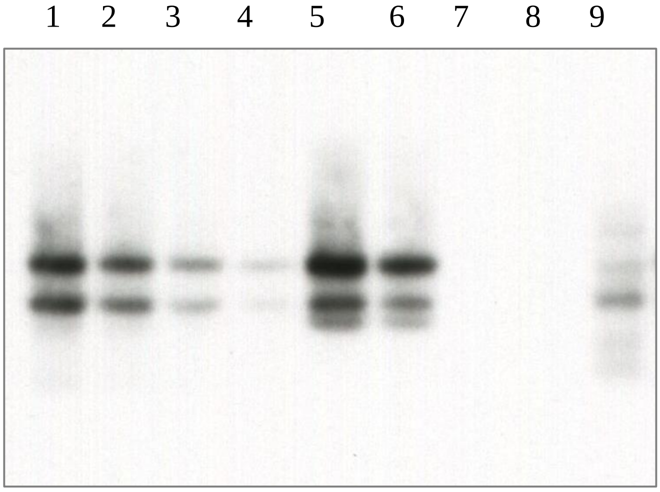

Order of samples

① S-4 (10 µl)
② S-4 (5 µl
③ S-4 (2.5 µl)
④ S-4 (1 µl)
⑤ Tl-om1 (10 µl)
⑥ TL-om1 (5 µl)
⑦ HIV cell line (U1) (10 µl)
⑧ Normal PBMC (10 µl)
⑨ S-1 (10 µl)

LTR-specific probe (7 5-bp)
TGTGTACTAAATTTCTCTCCTGAGAGTGCTA
TAGGATGGGCTGTCGCTGGCTCCGAGCCAA
CGGAGTCGCCGGTA

C

Detected by clone-specific probe

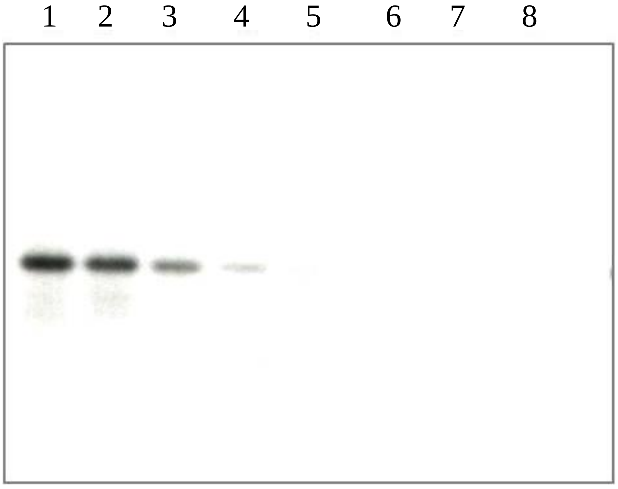

Order of samples

① S-4 (10 µl)
② S-4 (5 µl
③ S-4 (2.5 µl)
④ S-4 (1 µl)
⑤ Tl-om1 (10 µl)
⑥ TL-om1 (5 µl)
⑦ HIV cell line (U1) (10 µl)
⑧ Normal PBMC (10 µl)

Blue clone-specific probe (171-bp)
(chrx:83705328 (-))
GGTGAGATTGCTTTCTTGTAGGCAGTATAT
AGTGGAGTGATGGTTTTTTTTGTTGTTGTC
CATTTAGCCAGTCTATATATTTTAAGTGGAA
AGTTTAATTCATTTATATTCAAAATCATAAT
TGATATGTGAATATTTATTCCTGTCATTTTA
CTAGTTGATTTCTGGTGG

D

Sample	Shear site	Tag system	PCR- southern	Comment
S-1	**209**	393	A smear-like pattern	All three approaches showed similar results
S-4	**222**	2675	A strong monoclonal band	Tag system and PCR-southern : consistent results<br>Shear sites vs. PCR-southern: controversial results

Approach	Blue vs. Red	Proportion of Blue vs. Red
Shear sites	222 vs. 87	2.6 vs.1
Tag system	2675 vs. 209	12.8 vs. 1

Also see Figure 1 and 2
for data of shear sites and tags

Supporting Table 1

Information on integration site positions of top-10 clones for each sample

		Chromosome	Strand	Position	A representative sequence for each corresponding integration site position
Top 10 clones of S-1	1	chr11	+	41829319	AATATCTAGTTAAAGGAGGCTGTGAGAATTAGAAAATATA
	2	chr11	-	37042565	GAAATGCATAGCACTAAATGCTCACAAGAGAAAGCAGGAA
	3	chr7	-	121751243	CATAGTTATAAAAACCACTTTACAATGTTCATCTCATACT
	4	chr13	+	69268469	GGAGACCTTTTATCTTTTCTTTTATAATCACTTAATGGTA
	5	chr18	-	46701081	CTTTCTCACCCTTTCTAGTTAGTAAAATCCTAGGGAATAT
	6	chr17	+	18847529	AATTCATAATTACCAAAACTTTGGTAGATGTCCTTAAGTA
	7	chr15	-	37836845	TTTAGCTTCTACTTATAAGTGAAAACATGCAGTATTTGAT
	8	chr2	-	100184973	ACCCTTGGCTTGGTCCCAGGATCAAATCTCTTTTCAAAAA
	9	chr6	+	10852456	GAATAATATGTTGAAGAGTTTTGGATTTTACCTTTCTTAA
	10	chr8	-	35831701	CTGAGCAACGTATCTTTCTCTTCTTTAAACTTTGAGTTTT
Top 10 clones of S-2	1	chr15	+	59364370	GCTATGACTGATGAAAGTGGTGGTACCTAAAGATTGGGGT
	2	chr13	-	74706141	TGTAGAAGCATAGTGGAAATAGGATGTTGAAGGACAGACG
	3	chr3	+	28073332	AAATCAATGTTCTAAGATATGTACTAAGTGCTAGGGAACA
	4	chr21	-	44242161	CAAGCTCACTGATGTTTTCTTCTGTTTGATCAATTCTGCT
	5	chr18	-	38428907	GATTATTAGTATGATAATTACTTTACCAATCTGGTTGCAG
	6	chrX	-	107427783	CAAAAGAGTCCAAACACTAAAGCAACCTTGCTTTATAACA
	7	chr13	-	84177236	CACAGGTTCTAGGAATTAGTGTGTGGATATCTTTGTGGGG
	8	chr21	+	25834766	ATACTCCTGTTCAGGGAAAAATTTGAGCCGGTTTTCAGCA
	9	chr2	+	234346116	GACTCCGATGGTGGGTACCACACATGCTTATCCTTCTCAT
	10	chr7	-	99740574	AGTTTTCAACCCAGTACAGGGACTGTTACATAGCATCTTC
Top 10 clones of S-3	1	chr4	-	563543	CATTGTTTGT GTACCTATGT ACCAGCCTTTTCAAATGAGG
	2	chr20	+	58007381	TATGTTTCCT TACATTACTT ACTAATAGTA ATAAATAGCA
	3	chr5	+	62579369	GTCTCAGATTCCATGCTCTGAGAAAAGTGTGTATGAATTT
	4	chr6	+	133958124	GGTTTTTTTTTTTTTTTTTTTTTTTTTGCCATTGTAGGAT
	5	chr3	-	126392282	GTTAACCTCTTGAATTTTGAGAACTAAGGTTAGATGCCTG
	6	chr3	+	178928610	ACTAGGTAATAAAGTCATGAACTAAACAGACCTTCATTGA
	7	chr8	+	119096533	ATGTCCCAGATGCTACCTCCTGGGATCAACTGCAAGTCGT
	8	chr10	-	111698526	CTCCATGGTCTTCCCTGAACACCTCCTACTGCCCTGCAAC
	9	chr13	+	21355493	AAAAGGTCTAGCCGTTGCAACTCAGTGGCATCCCCATCAC
	10	chr18	+	62126326	CATAATCACTTTAAATGTGATTGGAATAAATTCTCCAGTT
Top 10 clones of S-4	1	chrX	-	83705328	CCTTTATAGGTGAGATTGCTTTCTTGTAGGCAGTATATAG
	2	chr14	+	30655896	AAAACTAAGTTTCAGCTCACAGTATTAGAGTGGGTTACAT
	3	chr14	+	49676335	GTGACTCAAAACAAAAAACAACACACTTACAGTCTTTTTA
	4	chr6	-	85461536	GAAGTTAACACTGATCTCTAATTAGTAAAGCTGTAGACTC
	5	chr16	-	17339636	CATTGTATCCTTCAGTCACCCATGAGAGATTGGATTTAGG
	6	chr8	+	96129917	ACTAGGCTGTGGACAAAAATGACATATGTCTCTTCCGGGC
	7	chr1	+	4032445	GGTTTCTAAAAGAATAGGTGCAAGTCTGTCATTGTGCTAA
	8	chr7	+	140001929	CACTTTCCCCATTGATGGTTGTGACACTTAAGCCCTCTTG
	9	chr21	+	35571080	CGGTGAGACCCCTGAAATACGAGTCATCCCCACTCCTGAC
	10	chr1	-	56007274	GGACACTTACTGTGAATTAGCTTGCAGGACTGGAAGTTGC
